# Lysosomal acid lipase, CSF1R, and PD-L1 determine functions of CD11c^+^ myeloid-derived suppressor cells

**DOI:** 10.1172/jci.insight.156623

**Published:** 2022-09-08

**Authors:** Ting Zhao, Sheng Liu, Xinchun Ding, Erica M. Johnson, Nasser H. Hanna, Kanhaiya Singh, Chandan K. Sen, Jun Wan, Hong Du, Cong Yan

**Affiliations:** 1 Department of Pathology and Laboratory Medicine,; 2 IU Simon Comprehensive Cancer Center,; 3 Department of Medical and Molecular Genetics, and; 4 Indiana Center for Regenerative Medicine and Engineering, Indiana University School of Medicine, Indianapolis, Indiana, USA.

**Keywords:** Immunology, Cancer immunotherapy

## Abstract

Lysosomal acid lipase (LAL) is a key enzyme in the metabolic pathway of neutral lipids. In the blood of LAL-deficient (*Lal^–/–^*) mice, increased CD11c^+^ cells were accompanied by upregulated programmed cell death ligand 1 (PD-L1) expression. Single-cell RNA sequencing of *Lal^–/–^* CD11c^+^ cells identified 2 distinctive clusters with a major metabolic shift toward glucose utilization and reactive oxygen species overproduction. Pharmacologically blocking pyruvate dehydrogenase in glycolysis not only reduced CD11c^+^ cells and their PD-L1 expression but also reversed their capabilities of T cell suppression and tumor growth stimulation. Colony-stimulating factor 1 receptor (CSF1R) played an essential role in controlling *Lal^–/–^* CD11c^+^ cell homeostasis and function and PD-L1 expression. Pharmacological inhibition of LAL activity increased CD11c, PD-L1, and CSF1R levels in both normal murine myeloid cells and human blood cells. Tumor-bearing mice and human patients with non–small cell lung cancer also showed CD11c^+^ cell expansion with PD-L1 and CSF1R upregulation and immunosuppression. There were positive correlations among CD11c, PD-L1, and CSF1R expression and negative correlations with LAL expression in patients with lung cancer or melanoma using The Cancer Genome Atlas database and patient samples. Therefore, CD11c^+^ cells switched their functions to immune suppression and tumor growth stimulation through CSF1R/PD-L1 upregulation and metabolic reprogramming.

## Graphical abstract

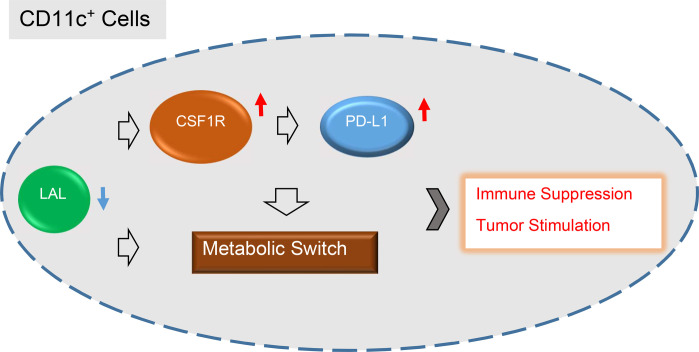

## Introduction

Immune cells in the tumor microenvironment actively participate in cancer initiation, progression, and metastasis (1). Thus, interactions between immune cells and tumor cells determine the balance between immunity and tolerance to tumor cells. The signaling pathway of programmed cell death ligand 1 (PD-L1, B7-H1, or CD274) and its receptor PD-1 (CD279) is an important mechanism tumors use to escape antitumor immune responses (2, 3) and maintain an immunosuppressive state in the tumor environment (4). PD-L1 binds to PD-1 or CD80 (B7-1) on activated T cells to mediate an inhibitory signal and to prevent the immune system from rejecting the tumor (5, 6). Antibody-based therapeutics targeting PD-L1 have shown clinical responses in multiple tumor types (7, 8). PD-L1 is broadly expressed in hematopoietic cells, including dendritic cells (DCs) (9). Blockade of PD-L1 on DCs has been reported to enhance T cell activation and cytokine production (10). However, it is still not entirely clear how PD-L1 expression is regulated in immune cells to affect tumor progression. DCs are a diverse group of specialized antigen-presenting cells (APCs) with key roles in the initiation and regulation of innate and adaptive immune responses. Much interest in modulating DC function has been given to cancer immunotherapy by generations of DC-based vaccines (11–13). Most DCs express CD11c as a surface marker (12, 14). The diversity and functions of CD11c^+^ cell subsets that are shaped by pathogenic conditions are not completely understood, especially those CD11c^+^ cells that tolerate and stimulate cancer growth.

Lysosomal acid lipase (LAL) is a key enzyme in the metabolic pathway of neutral lipids, which hydrolyzes cholesteryl esters and triglycerides in the lysosome of cells to generate free fatty acids and cholesterol. Clinical case reports indicate that mutations of the LAL gene are associated with carcinogenesis in patients (15). In LAL-knockout (*Lal^–/–^*) mice, myeloid populations systemically expand from hematopoietic progenitors in the bone marrow, suppress T cell proliferation and function, and stimulate tumor growth and invasion in both syngeneic and allogeneic backgrounds (16–18). The LAL deficiency–induced immunocompromise also allows growth of human cancer cells in mice (19).

The present study found that LAL deficiency in the *Lal^–/–^* mouse model increased the CD11c^+^ cell population in the blood that was associated with upregulation of PD-L1 expression. These *Lal^–/–^* CD11c^+^ cells shared characteristics of myeloid-derived suppressor cells (MDSCs) by suppressing T cells and stimulating tumor growth. Single-cell RNA sequencing (scRNA-Seq) analysis identified 2 distinct clusters with differential gene expression patterns, and revealed a major metabolic shift toward glucose utilization and reactive oxygen species (ROS) overproduction in *Lal^–/–^* CD11c^+^ cells. Colony-stimulating factor 1 receptor (CSF1R) has been identified as a gatekeeper in controlling *Lal^–/–^* CD11c^+^ cell homeostasis and functions. Findings in CD11c^+^ cells of *Lal^–/–^* mice were reproduced in CD11c^+^ cells of tumor-bearing mice and human cancer patients. In humans, there were positive correlations among *CD11C*, *CD274* (PD-L1), *CSF1R*, and *IFNG* (IFN-γ) expression and negative correlations with *LIPA* (LAL) expression in multiple cancer forms using The Cancer Genome Atlas (TCGA) database. Human patients with non–small cell lung cancer showed CD11c^+^ cell expansion with PD-L1 and CSF1R upregulation and decreased LAL expression in their blood. These studies demonstrate that in addition to their APC function, CD11c^+^ cells are able to switch to subpopulations that exhibit immune suppression and tumor growth stimulation in the tumor environment as a result of metabolic reprogramming and PD-L1 and CSF1R upregulation.

## Results

### Increase of PD-L1 expression in Lal^–/–^ CD11c^+^ cells.

**Figure 1:**
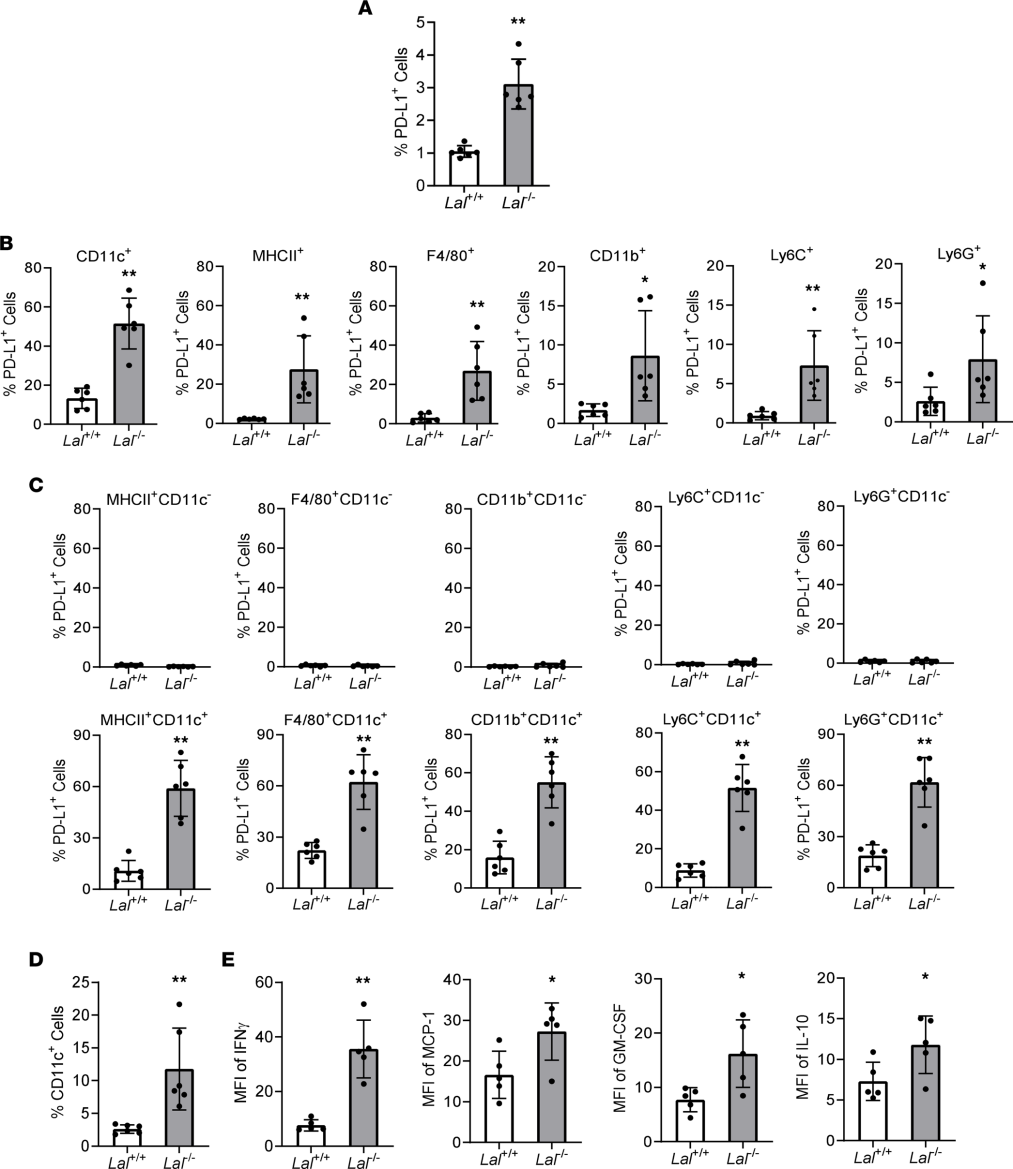
PD-L1 expression is increased in *Lal^–/–^* CD11c^+^ cells. (**A**) Percentage of PD-L1^+^ cells in the blood of *Lal^+/+^* and *Lal^–/–^* mice by flow cytometry analysis. (**B**) PD-L1 expression in blood CD11c^+^, MHCII^+^, F4/80^+^, CD11b^+^, Ly6C^+^, and Ly6G^+^ cells of *Lal^–/–^* versus *Lal^+/+^* mice by flow cytometry analysis. (**C**) PD-L1 expression in CD11c^−^ or CD11c^+^ double-gated myeloid cells of *Lal^–/–^* versus *Lal^+/+^* blood by flow cytometry analysis. (**D**) Percentage of CD11c^+^ cells in the blood of *Lal^+/+^* and *Lal^–/–^* mice by flow cytometry analysis. (**E**) Cytokine expression in *Lal^–/–^* versus *Lal^+/+^* CD11c^+^ cells by flow cytometry analysis. Data are expressed as mean ± SD. Experiments were independently repeated, *n* = 6 for **A**–**D**, *n* = 5 for **E**. *P < 0.05, **P < 0.01, unpaired Student’s *t* test.

The percentage of PD-L1^+^ cells in the whole blood was increased in *Lal^–/–^* mice versus *Lal*^+/+^ mice (Figure 1A and Supplemental Figure 1A; supplemental material available online with this article; https://doi.org/10.1172/jci.insight.156623DS1). Since T cells in the blood of *Lal^–/–^* mice were dramatically reduced compared with those of *Lal^+/+^* mice (20), it is unlikely that the T cells are responsible for the increase of PD-L1 expression. To further assess which cell population in the myeloid component is responsible for the increase of PD-L1 expression, the PD-L1 level in various myeloid cell subtypes was analyzed by flow cytometry. Compared with *Lal^+/+^* mice, PD-L1 expression was upregulated in CD11c^+^, MHCII^+^, F4/80^+^, CD11b^+^, Ly6C^+^, and Ly6G^+^ myeloid cells from the blood of *Lal^–/–^* mice when a single surface marker was used (Figure 1B). However, further analyses demonstrated that all the CD11c^–^ double-gated myeloid subtypes, including CD11b^+^CD11c^−^, MHCII^+^CD11c^−^, F4/80^+^CD11c^−^, Ly6C^+^CD11c^−^, and Ly6G^+^CD11c^−^ cells, showed no increase in PD-L1 expression, while all the CD11c^+^ double-gated myeloid subtypes, including CD11b^+^CD11c^+^, MHCII^+^CD11c^+^, F4/80^+^CD11c^+^, Ly6C^+^CD11c^+^, and Ly6G^+^CD11c^+^ cells, showed increased PD-L1 expression in the blood of *Lal^–/–^* mice versus *Lal^+/+^* mice (Figure 1C). An example of PD-L1 expression in CD11b^+^CD11c^−^ and CD11b^+^CD11c^+^ cells and their gating strategy are shown in Supplemental Figure 1B. This suggests that LAL deficiency–induced PD-L1 expression was restricted to the CD11c^+^ myeloid subpopulation, whose percentage was increased in the blood of *Lal^–/–^* mice versus *Lal^+/+^* mice (Figure 1D and Supplemental Figure 1C). Flow cytometry analysis also showed upregulation of MFI of IFN-γ, MCP-1, GM-CSF, and IL-10 proteins in *Lal^–/–^* CD11c^+^ cells versus *Lal^+/+^* CD11c^+^ cells (Figure 1E). The percentage differences are shown in Supplemental Figure 1, D and E, in which IFN-γ, MCP-1, GM-CSF, IL-10, IL-1β, and IL-2 show statistically significant differences. IFN-γ has been reported to regulate PD-L1 expression (21). To see whether IFN-γ produced by CD11c^+^ cells is essential for PD-L1 expression, *Lal^+/+^* or *Lal^–/–^* CD11c^+^ cells were treated with IFN-γ–neutralizing antibody or control IgG for 2 days. The percentage of PD-L1^+^ cells was decreased in CD11c^+^ cells with IFN-γ–neutralizing antibody treatment (Supplemental Figure 1F), suggesting that CD11c^+^ cell–derived IFN-γ participates in regulating PD-L1 expression.

### Lal^–/–^ CD11c^+^ cells suppress T cell proliferation and stimulate tumor growth through PD-L1.

**Figure 2:**
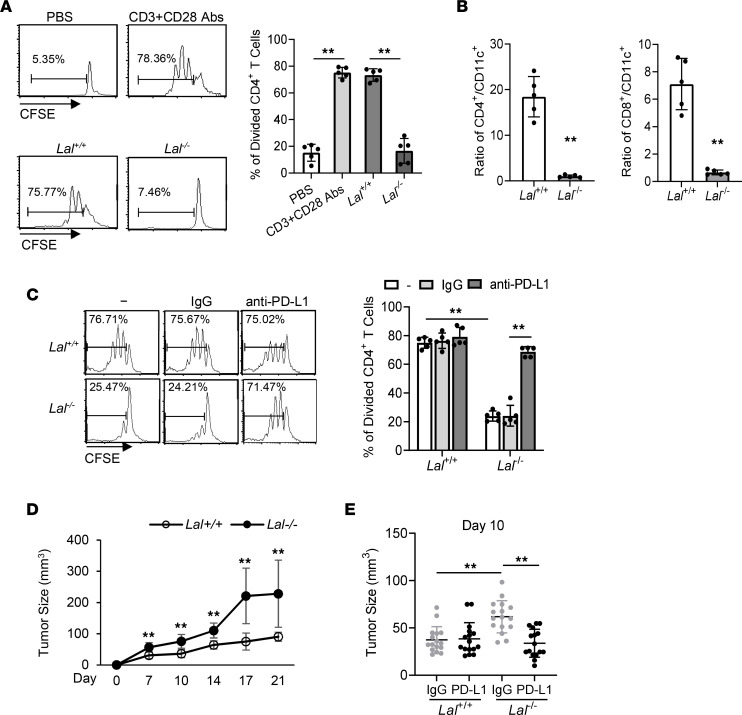
*Lal^–/–^* CD11c^+^ cells suppress T cell proliferation and stimulate tumor cell growth through PD-L1. (**A**) CFSE-labeled *Lal^+/+^* CD4^+^ T cells were stimulated with anti-CD3 mAb plus anti-CD28 mAb for 4 days in the presence or absence of *Lal^+/+^* or *Lal^–/–^* CD11c^+^ cells at a 1:1 CD4^+^ T cell/CD11c^+^ cell ratio. Proliferation of labeled CD4^+^ T cells was analyzed by flow cytometry. Peaks represent cell division cycles. PBS was used as a negative control. Left: A representative CFSE dilution by flow cytometry. Right: Statistical analyses of percentage of divided CD4^+^ T cells. (**B**) Ratios of CD4^+^ T cells to CD11c^+^ cells and CD8^+^ T cells to CD11c^+^ cells in the blood of *Lal^–/–^* versus *Lal^+/+^* mice were analyzed by flow cytometry analysis. (**C**) Freshly isolated *Lal^+/+^* or *Lal^–/–^* CD11c^+^ cells were pretreated with IgG or anti–PD-L1 antibody (5 μg/mL), then cocultured with CFSE-labeled *Lal^+/+^* CD4^+^ T cells (at 1:1 ratio) for T cell proliferation assay as described for **A**. (**D**) B16 melanoma cells (2 × 10^5^) were mixed with *Lal^+/+^* or *Lal^–/–^* CD11c^+^ cells (2 × 10^5^) and injected subcutaneously at the flank region of *Lal^+/+^* recipient mice. Tumor size was measured at 7, 10, 14, 17, and 21 days after cell injection and determined using the formula (length × width^2^)/2. (**E**) *Lal^+/+^* or *Lal^–/–^* CD11c^+^ cells were pretreated with IgG or anti–PD-L1 antibody (5 μg/mL), then coinjected with B16 melanoma cells into the flank region of *Lal^+/+^* recipient mice. Data are expressed as mean ± SD. Experiments were independently repeated, *n* = 5 for **A**–**C**, *n* = 10 for **D**, *n* = 16 for **E**. **P < 0.01, 1-way ANOVA for **A** and **E**, unpaired Student’s *t* test for **B** and **D**, 2-way ANOVA for **C**.

We characterized the functions of *Lal^–/–^* CD11c^+^ cells. First, freshly isolated *Lal^+/+^* or *Lal^–/–^* CD11c^+^ blood cells were cocultured with wild-type splenic CD4^+^ T cells for CFSE proliferation assay in vitro, in which *Lal^–/–^* CD11c^+^ cells drastically suppressed CD4^+^ T cell proliferation versus *Lal^+/+^* CD11c^+^ cells (Figure 2A). The in vivo ratios of CD4^+^ T cells to CD11c^+^ cells and CD8^+^ T cells to CD11c^+^ cells were also greatly decreased in the blood of *Lal^–/–^* mice (Figure 2B). To evaluate whether the upregulated PD-L1 expression in *Lal^–/–^* CD11c^+^ cells is responsible for the suppression of T cell proliferation, CFSE-labeled T cells were cocultured with isolated *Lal^+/+^* or *Lal^–/–^* CD11c^+^ cells that were pretreated with control IgG or anti–PD-L1 antibody. Results showed that blocking PD-L1 with anti–PD-L1 antibody reversed the *Lal^–/–^* CD11c^+^ cells’ suppressive activity on T cell proliferation (Figure 2C).

Next, isolated *Lal^+/+^* or *Lal^–/–^* CD11c^+^ cells were subcutaneously coinjected with B16 melanoma cells into *Lal^+/+^* recipient mice at flank sites. Compared with *Lal^+/+^* CD11c^+^ cells, *Lal^–/–^* CD11c^+^ cells stimulated tumor growth in vivo (Figure 2D). However, after pretreatment with anti–PD-L1 antibody, *Lal^–/–^* CD11c^+^ cells’ stimulation of tumor growth was reversed (Figure 2E).

### Characteristics of Lal^–/–^ versus Lal^+/+^ CD11c^+^ cells by scRNA-Seq.

**Figure 3:**
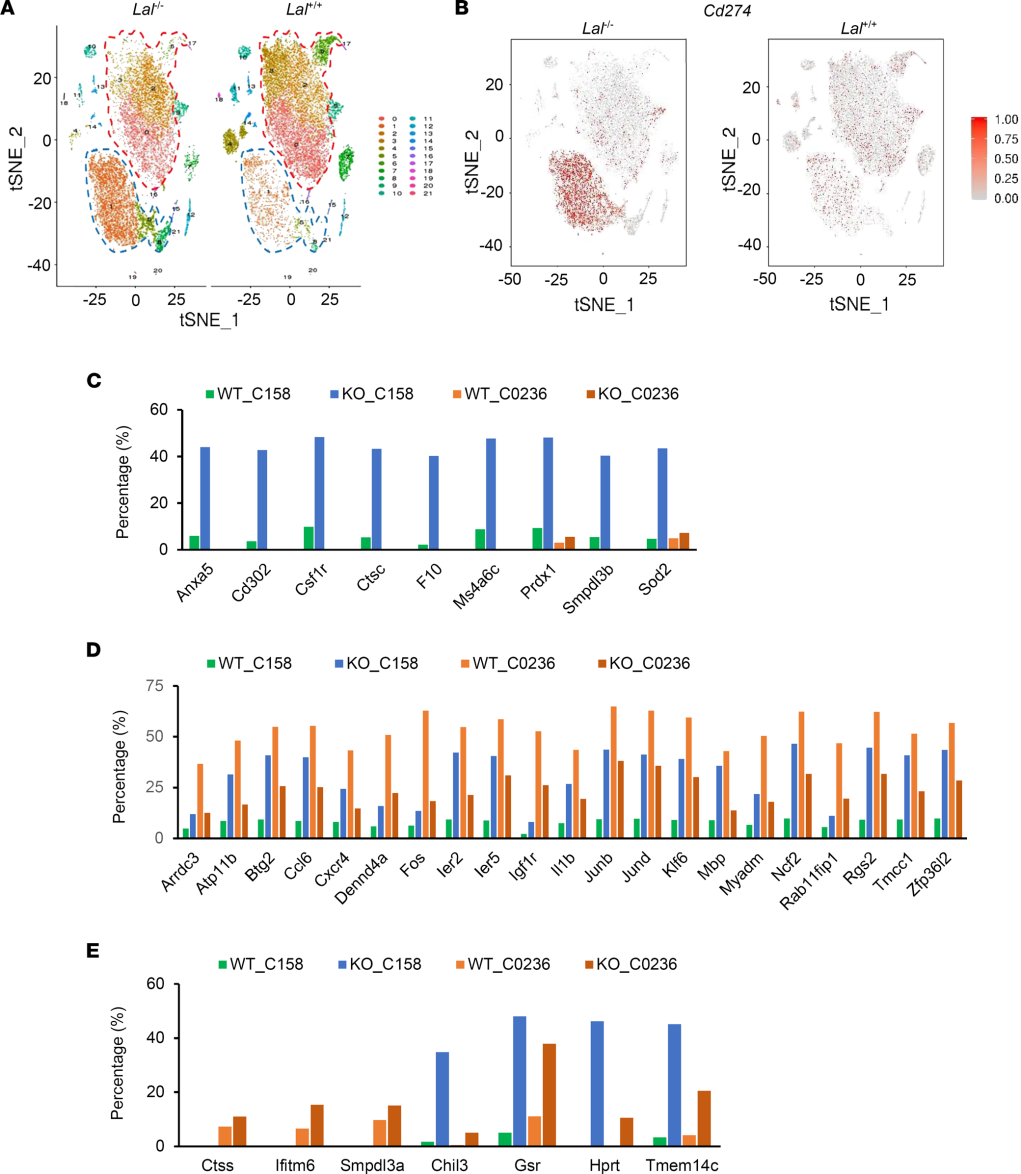
Identification and gene expression of *Lal^–/–^* versus *Lal^+/+^* CD11c^+^ cell clusters by scRNA-Seq. (**A**) t-SNE plot of CD11c^+^ cell clusters from *Lal^–/–^* versus *Lal^+/+^* mice. Each dot represents a single cell colored by cluster assignment. The dashed blue line circles cluster 158, and the dashed red line circles cluster 0236. (**B**) Feature plot of *Cd274* (PD-L1) expression across cell clusters identified in **A**. (**C**) Percentages of cells for expressed genes were increased in cluster 158 and relatively unchanged in cluster 0236 of *Lal^–/–^* versus *Lal^+/+^* CD11c^+^ cells. The percentage was calculated using the number of expressed cells for the gene divided by the number of cells for this sample. (**D**) Percentages of cells for expressed genes were increased in cluster 158 but decreased in cluster 0236 of *Lal^–/–^* versus *Lal^+/+^* CD11c^+^ cells. (**E**) Percentages of cells for expressed genes were increased in cluster 0236 but undetectable or increased in cluster 158 of *Lal^–/–^* versus *Lal^+/+^* CD11c^+^ cells.

Since the effects of *Lal^–/–^* CD11c^+^ cells (e.g., T cell suppression and tumor stimulation) were the opposite of the effects of normal DCs (e.g., T cell stimulation and tumor suppression), we sought to reveal the intrinsic molecular transition and get a more comprehensive understanding of *Lal^–/–^* versus *Lal^+/+^* CD11c^+^ cells. scRNA-Seq is a powerful technique to clarify heterogeneity of the immune system by identifying novel distinct immune cell subsets and building trajectories for immune cells (22). CD11c^+^ cells were isolated from the blood of *Lal^+/+^* and *Lal^–/–^* mice for scRNA-Seq analysis. t-Distributed stochastic neighbor embedding (t-SNE) plot analysis identified 2 major distinctive cellular clusters of CD11c^+^ cells: (a) clusters 1, 5, 8 (referred to as cluster 158 hereafter) with increased cellular numbers in *Lal^–/–^* CD11c^+^ cells versus *Lal^+/+^* CD11c^+^ cells, and (b) clusters 0, 2, 3, 6 (referred to as cluster 0236 hereafter) with decreased cellular numbers in *Lal^–/–^* CD11c^+^ cells versus *Lal^+/+^* CD11c^+^ cells (Figure 3A). Cluster 158 demonstrated the monocyte feature, while cluster 0236 demonstrated the neutrophil feature (Supplemental Table 1). Interestingly, there were significantly more cells expressing *Cd274* (the gene encoding PD-L1) in cluster 158, and fewer in cluster 0236, of *Lal^–/–^* versus *Lal^+/+^* CD11c^+^ cells (Figure 3B). There were more cells expressing *Adgre1*, *Ly6c1*, *Ly6g*, *Ifng*, *Ccl2*, *Csf2*, and *Il10* (the genes encoding F4/80, Ly6C, Ly6G, IFN-γ, MCP-1, GM-CSF, and IL-10, respectively) in cluster 158 of *Lal^–/–^* versus *Lal^+/+^* CD11c^+^ cells (Supplemental Figure 2). Other top 50 upregulated and top 50 downregulated genes in cluster 158 and cluster 0236 of *Lal^–/–^* CD11c^+^ cells can be categorized into 3 groups: (a) those expressed by an increased number of cells in cluster 158 with no change in cluster 0236 (Figure 3C); (b) those expressed by an increased number of cells in cluster 158 and a decreased number in cluster 0236 (Figure 3D); and (c) those expressed by an increased number of cells in cluster 0236 with no change or an increase in cluster 158 (Figure 3E). Log fold change, gene expression, and cellular numbers of the top 50 upregulated and downregulated genes among all clusters, cluster 158, and cluster 0236 are presented in Supplemental Tables 2–7.

### Metabolic reprogramming in Lal^–/–^ CD11c^+^ cells.

**Figure 4:**
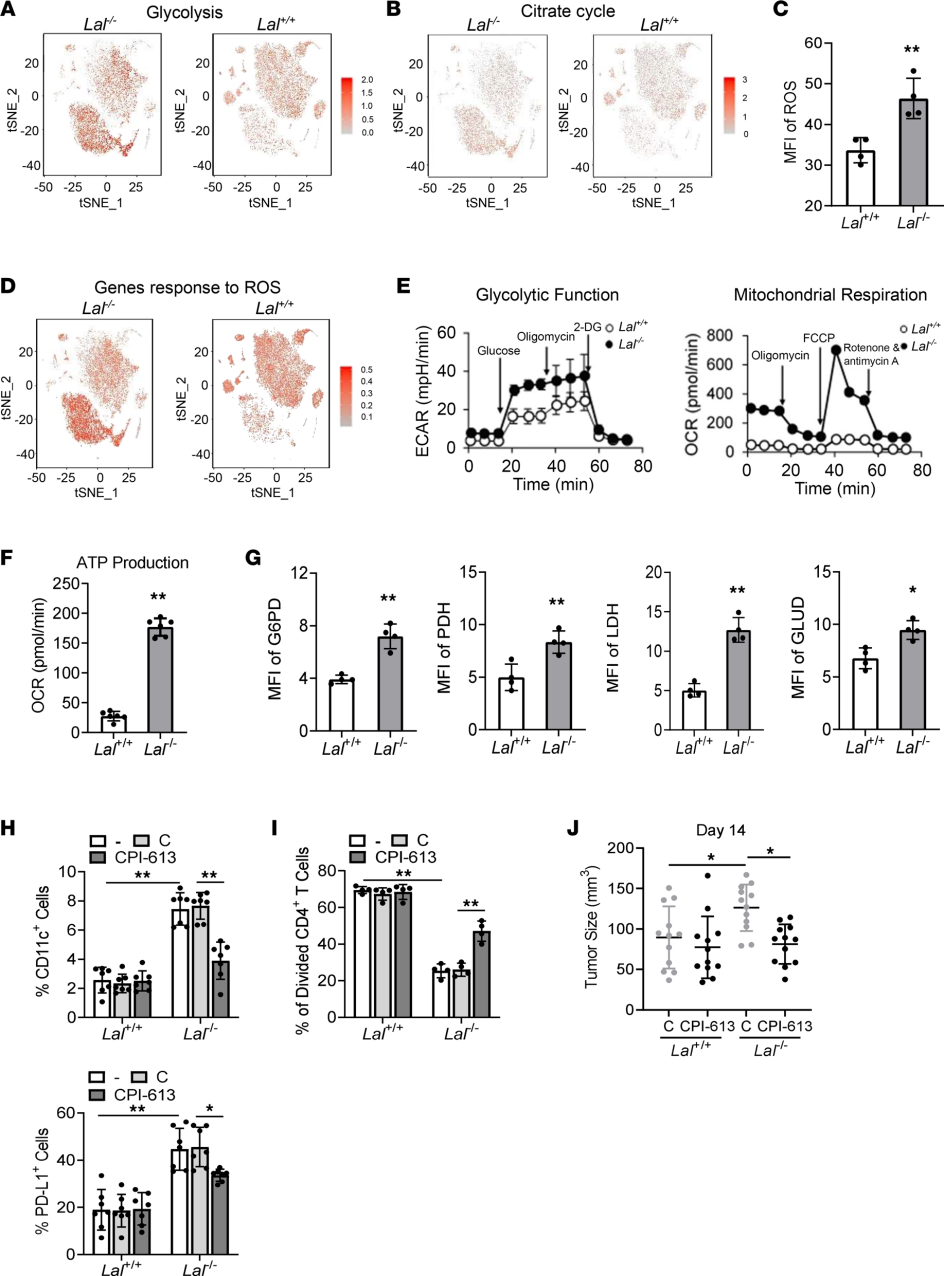
Metabolic reprogramming in *Lal^–/–^* CD11c^+^ cells. (**A** and **B**) Gene expression of glycolysis (**A**) and citrate cycle (**B**) across cell clusters in t-SNE plots of CD11c^+^ cells from *Lal^–/–^* versus *Lal^+/+^* mice. (**C**) Statistical analysis of ROS MFI in *Lal^–/–^* versus *Lal^+/+^* CD11c^+^ cells by flow cytometry. (**D**) Expression of gene response to ROS across cell clusters in t-SNE plots of CD11c^+^ cells from *Lal^–/–^* versus *Lal^+/+^* mice. (**E**) Extracellular acidification rate (ECAR) of glycolysis and oxygen consumption rate (OCR) in mitochondrial respiration in *Lal^–/–^* versus *Lal^+/+^* CD11c^+^ cells. (**F**) ATP production in mitochondrial respiration in *Lal^–/–^* versus *Lal^+/+^* CD11c^+^ cells. (**G**) MFI of G6PD, PDH, LDH, and GLUD expression in *Lal^–/–^* versus *Lal^+/+^* CD11c^+^ cells by flow cytometry. (**H**) Percentage of CD11c^+^ cells in the blood and percentage of PD-L1^+^ cells in CD11c^+^ blood cells after CPI-613 treatment by flow cytometry analysis. (**I**) CPI-613–pretreated CD11c^+^ cells were cocultured with CFSE-labeled *Lal^+/+^* CD4^+^ T cells for T cell proliferation assay. (**J**) CPI-613–pretreated CD11c^+^ cells (2 × 10^5^) were coinjected with B16 melanoma cells (2 × 10^5^) into the flank region of *Lal^+/+^* recipient mice for tumor growth assay. Data are expressed as mean ± SD. Experiments were independently repeated, *n* = 4 for **C**, **G**, and **I**, *n* = 6–8 for **E** and **F**, *n* = 7 for **H**, *n* = 12 for **J**. *P < 0.05, **P < 0.01, unpaired Student’s *t* test for **C**, **F**, and **G**, 2-way ANOVA for **H** and **I**, 1-way ANOVA for **J**.

Metabolic regulation is important to myeloid differentiation and function (23–27). LAL deficiency reduces fatty acid oxidation, which leads to metabolic reprogramming of *Lal^–/–^* CD11c^+^ cells. Veritably, t-SNE plots and pathway analysis of scRNA-Seq showed that genes involved in glycolysis and the citrate cycle were upregulated in *Lal^–/–^* versus *Lal^+/+^* cluster 158 of CD11c^+^ cells (Figure 4, A and B; and Supplemental Figure 3, A–C), which overlaps with the increased *Cd274* expression in t-SNE plots (Figure 3B). This metabolic reprogramming was associated with ROS overproduction (Figure 4C and Supplemental Figure 3D). When expression of genes responding to ROS (selected from the Mouse Genome Informatics database) was compared between these 2 clusters, there was a significant shift from cluster 0236 to cluster 158 (Figure 4D). This observation was confirmed by trajectory analysis (Supplemental Figure 3E), in which trajectory dynamic changes and differential lineages were clearly different in cluster 158 and cluster 0236 of *Lal^–/–^* versus *Lal^+/+^* CD11c^+^ cells. Accordingly, scRNA-Seq analysis showed upregulation of the *Gsr* gene (coding for glutathione reductase, an important cellular antioxidant enzyme) in both cluster 158 and cluster 0236 of *Lal^–/–^* CD11c^+^ cells (Figure 3E and Supplemental Figure 3F). Seahorse studies showed that both glycolytic metabolism and oxidative phosphorylation in mitochondrial respiration were significantly increased in *Lal^–/–^* versus *Lal^+/+^* CD11c^+^ cells by extracellular acidification rate and oxygen consumption rate analyses (Figure 4E and Supplemental Figure 4, A and B). In addition, *Lal^–/–^* CD11c^+^ cells demonstrated a higher rate of ATP production than *Lal^+/+^* CD11c^+^ cells (Figure 4F). Protein expression of several key metabolic enzymes in glucose metabolism, including pyruvate dehydrogenase (PDH), glucose-6-phosphate dehydrogenase (G6PD), and lactate dehydrogenase (LDH), was significantly increased in *Lal^–/–^* CD11c^+^ cells compared with *Lal^+/+^* CD11c^+^ cells (Figure 4G and Supplemental Figure 4, C and D). Additionally, glutamate dehydrogenase (GLUD) in the glutamine pathway showed upregulation in *Lal^–/–^* CD11c^+^ cells at the protein level (Figure 4G and Supplemental Figure 4, C and D). These results suggest a metabolic switch to glucose and amino acid utilization in *Lal^–/–^* CD11c^+^ cells.

Interestingly, PD-L1 expression was subjected to metabolic switch regulation, as injection of the PDH inhibitor CPI-613 into *Lal^–/–^* mice reduced the CD11c^+^ cell population and PD-L1 expression in *Lal^–/–^* CD11c^+^ cells (Figure 4H). Furthermore, CPI-613–pretreated *Lal^–/–^* CD11c^+^ cells showed reduced capabilities of T cell suppression when cocultured with CD4^+^ T cells in vitro (Figure 4I) and tumor growth stimulation when coinjected with B16 melanoma cells subcutaneously in *Lal^+/+^* mice (Figure 4J). Clearly, blocking PDH in the glycolysis pathway impaired *Lal^–/–^* CD11c^+^ cells’ immunosuppressive and tumor stimulatory functions. However, CPI-613 treatment in *Lal^–/–^* CD11c^+^ cells did not change the IFN-γ expression level (Supplemental Figure 4E).

### CSF1R controls Lal^–/–^ CD11c^+^ cells’ PD-L1 regulation, T cell suppression, and tumor growth stimulation.

**Figure 5:**
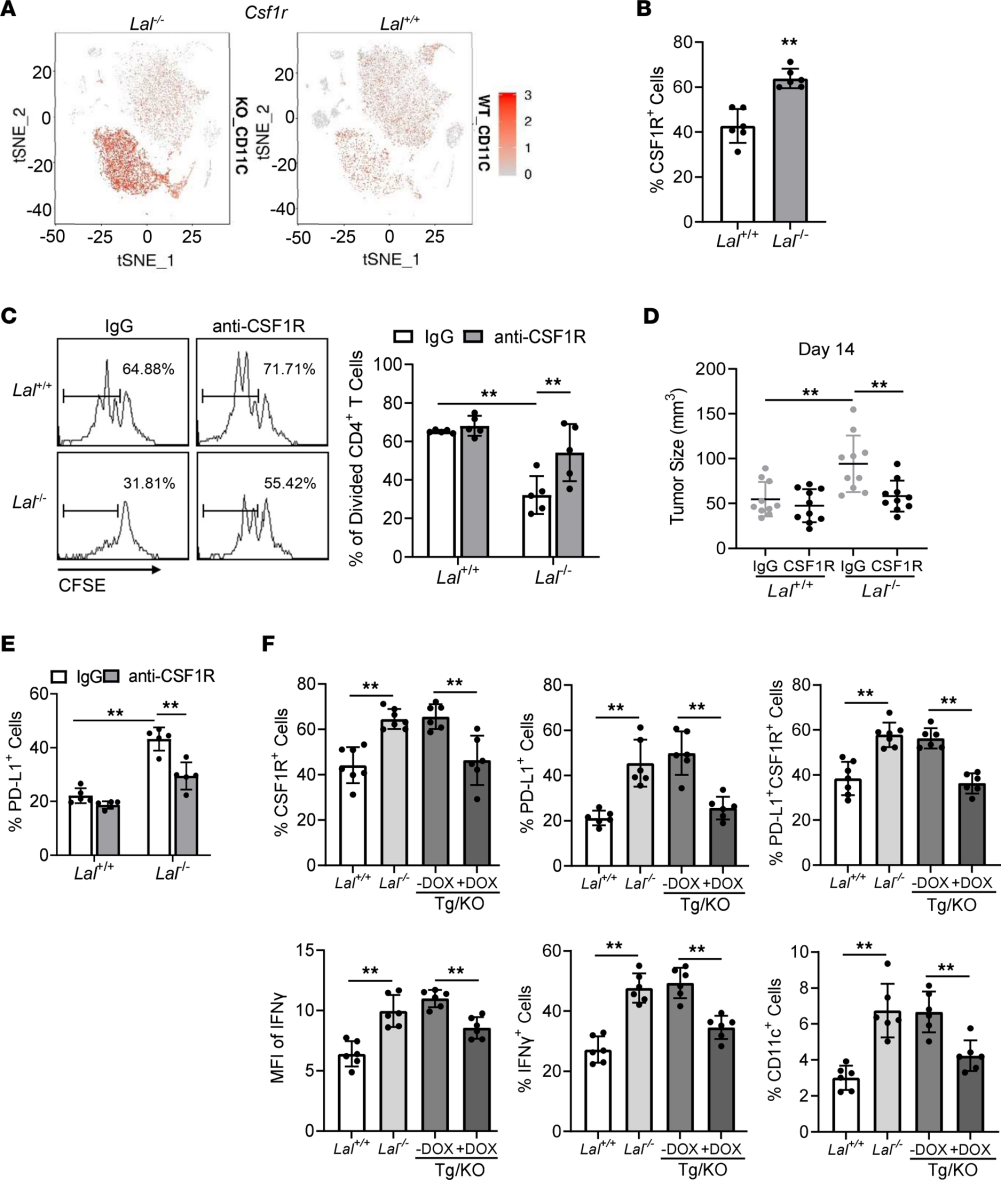
CSF1R expression and function in *Lal^–/–^* CD11c^+^ cells. (**A**) *Csf1r* expression across cell clusters in t-SNE plots of CD11c^+^ cells from *Lal^–/–^* versus *Lal^+/+^* mice. (**B**) Percentage of CSF1R^+^ cells in blood CD11c^+^ cells of *Lal^–/–^* versus *Lal^+/+^* mice by flow cytometry analysis. (**C**) Isolated *Lal^+/+^* or *Lal^–/–^* CD11c^+^ cells were pretreated with IgG or anti-CSF1R antibody (5 μg/mL) and cocultured with CFSE-labeled *Lal^+/+^* CD4^+^ T cells (at 1:1 ratio). The proliferation of labeled CD4^+^ T cells was analyzed by flow cytometry. (**D**) Isolated *Lal^+/+^* or *Lal^–/–^* CD11c^+^ cells (2 × 10^5^) were pretreated with IgG or anti-CSF1R antibody (5 μg/mL) and coinjected with B16 melanoma cells (2 × 10^5^) into the flank region of *Lal^+/+^* recipient mice. The tumor size was measured at 14 days after cell injection. (**E**) Percentage of PD-L1^+^ cells in CD11c^+^ cells after anti-CSF1R antibody treatment (5 μg/mL) by flow cytometry analysis. (**F**) Percentages of CSF1R^+^ cells, PD-L1^+^ cells, PD-L1^+^CSF1R^+^ cells, and IFN-γ^+^ cells and MFI of IFN-γ in blood CD11c^+^ cells and percentage of blood CD11c^+^ cells in *Lal^+/+^*, *Lal^–/–^*, untreated (–DOX), and DOX-treated (+DOX) *c-fms*–Tg/KO (Tg/KO) mice by flow cytometry analysis. Data are expressed as mean ± SD. Experiments were independently repeated, *n* = 6 for **B**, *n* = 5 for **C** and **E**, *n* = 10 for **D**, *n* = 6–7 for **F**. **P < 0.01, unpaired Student’s *t* test for **B**, 2-way ANOVA for **C** and **E**, 1-way ANOVA for **D** and **F**.

In searching for molecular signaling that controls PD-L1 upregulation in *Lal^–/–^* CD11c^+^ cells, CSF1R was selected based on scRNA-Seq analysis (Figure 3C), as it plays a critical role in myeloid genesis and promotes the differentiation of progenitors into heterogeneous populations of myeloid cells (28, 29). Importantly, t-SNE plot analysis of CD11c^+^ cells showed that the number of *Csf1r* gene–positive cells was increased in cluster 158 of *Lal^–/–^* CD11c^+^ blood cells (Figure 3C and Figure 5A), which overlaps with the increased *Cd274* expression (Figure 3B) and the metabolic switch (Figure 4, A and B). Flow cytometry analysis confirmed the increased level of CSF1R protein in the *Lal^–/–^* CD11c^+^ blood cells (Figure 5B and Supplemental Figure 5A). However, this was not the case in *Lal^–/–^* CD11b^+^Ly6G^+^ cells (Supplemental Figure 5B), a traditional *Lal^–/–^* MDSC population that also strongly suppresses T cell proliferation (16, 18, 30) and stimulates tumor growth (17). CSF1R expression was upregulated in CD11c^+^ myeloid cells from *Lal^–/–^* mice, including CD11b^+^CD11c^+^, Ly6C^+^CD11c^+^, Ly6G^+^CD11c^+^, MHCII^+^CD11c^+^, and F4/80^+^CD11c^+^ cells (Supplemental Figure 5C). To evaluate whether the upregulated CSF1R expression in *Lal^–/–^* CD11c^+^ cells is responsible for *Lal^–/–^* CD11c^+^ cell immunosuppressive and tumor stimulatory functions, isolated *Lal^+/+^* or *Lal^–/–^* CD11c^+^ cells were pretreated with control IgG or anti-CSF1R antibody, and then cocultured with splenic CD4^+^ T cells in vitro or subcutaneously coinjected with B16 melanoma cells in vivo. Results showed that blocking CSF1R reversed *Lal^–/–^* CD11c^+^ cells’ suppressive activity on T cell proliferation (Figure 5C) and impaired stimulation of tumor growth in mice (Figure 5D). Furthermore, treatment of *Lal^–/–^* mice with anti-CSF1R antibody decreased the percentage and MFI of PD-L1^+^ cells in CD11c^+^ blood cells (Figure 5E and Supplemental Figure 5D), suggesting that the PD-L1 level was regulated by CSF1R. In the T cell suppression study, the CSF1R ligand CSF1 came from both CD11c^+^ and CD4^+^ T cells. *Lal^–/–^* CD11c^+^ cells showed higher CSF1 production than *Lal^+/+^* CD11c^+^ cells, while CD4^+^ T cells showed lower production of CSF1 (Supplemental Figure 5E). However, the IFN-γ level was not significantly changed by blocking of CSF1R in *Lal^–/–^* CD11c^+^ cells (Supplemental Figure 5D). A *c-fms*–Tg/KO triple-transgenic mouse model with myeloid-specific doxycycline-inducible expression of human LAL (hLAL) (18) was used to test whether hLAL expression in myeloid cells corrects CSF1R and PD-L1 upregulation in CD11c^+^ cells. Indeed, in comparison with non-doxycycline-treated *c-fms*–Tg/KO triple-transgenic mice (–DOX group), myeloid-specific expression of hLAL (+DOX group) corrected the increased percentages and MFI of CSF1R^+^, PD-L1^+^, IFN-γ^+^, and PD-L1^+^CSF1R^+^ double-positive populations in *Lal^–/–^* CD11c^+^ cells and decreased the percentage of the CD11c^+^ cell population (Figure 5F and Supplemental Figure 5F). Therefore, myeloid expression of hLAL in the humanized model critically controls *Lal^–/–^* CD11c^+^ cell homeostasis and pathogenic functions through the CSF1R/PD-L1 axis.

### Pharmacological inhibition of LAL upregulates the expression of CD11c, PD-L1, and CSF1R in murine HD1A myeloid cells and human blood cells.

**Figure 6:**
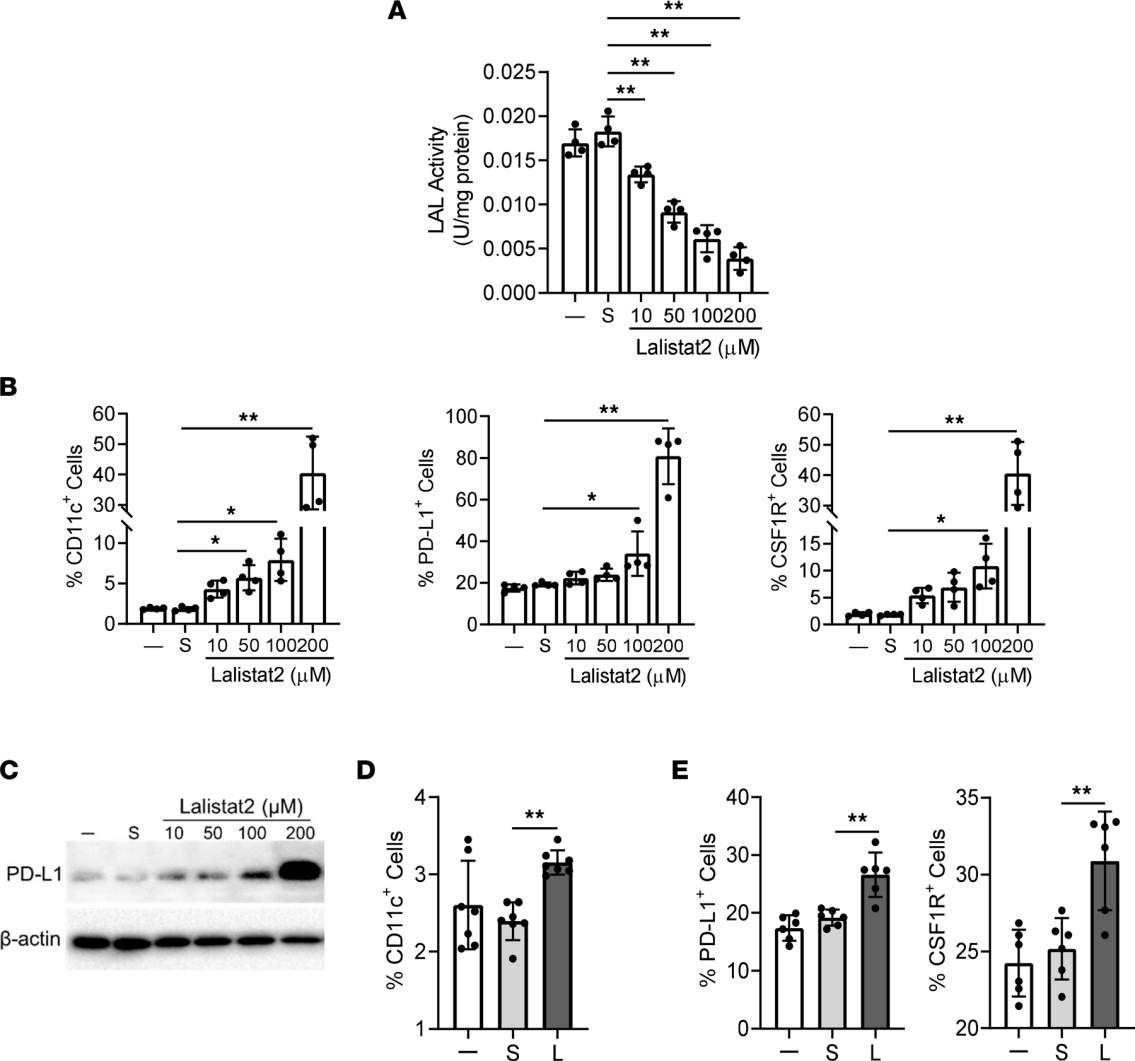
Expression of PD-L1 and CSF1R in mouse myeloid cells and human blood CD11c^+^ cells after Lalistat2 treatment. (**A**) LAL enzymatic activity in HD1A myeloid cells after incubation with 10 μM, 50 μM, 100 μM, and 200 μM Lalistat2 or DMSO (S) for 72 hours. (**B**) Murine HD1A myeloid cells were incubated with 10 μM, 50 μM, 100 μM, and 200 μM Lalistat2 or DMSO (S) for 72 hours. Percentages of CD11c^+^, PD-L1^+^, and CSF1R^+^ cells in HD1A myeloid cells were analyzed by flow cytometry. (**C**) Expression of PD-L1 in HD1A myeloid cells after Lalistat2 or DMSO treatment for 72 hours by Western blot analysis. Representative blots are shown. (**D**) Human white blood cells from healthy individuals were incubated with 10 μM Lalistat2 (L) or DMSO (S) for 24 hours. Percentages of CD11c^+^ cells in the whole white blood cells were analyzed by flow cytometry. (**E**) Percentages of PD-L1^+^ and CSF1R^+^ cells in blood CD11c^+^ cells of healthy individuals treated with Lalistat2 (L) versus DMSO (S). Data are expressed as mean ± SD. Experiments were independently repeated, *n* = 4 for **A** and **B**, *n* = 3 for **C**, *n* = 6 for **D**, *n* = 5 for **E**. *P < 0.05, **P < 0.01, 1-way ANOVA.

The above observations were based on the genetic loss of LAL in mice. To confirm these findings by pharmacological inhibition, the LAL-specific inhibitor Lalistat2 (31, 32) was used to treat the previously established murine myeloid cell line HD1A (33). The LAL enzymatic activity in HD1A myeloid cells was inhibited by Lalistat2 in a dose-dependent fashion (Figure 6A). As Figure 6B and Supplemental Figure 6A show, Lalistat2 treatment significantly increased the percentages of CD11c^+^, PD-L1^+^, and CSF1R^+^ cells in a concentration-dependent fashion in HD1A cells. Western blot analysis confirmed the upregulation of PD-L1 in HD1A cells after Lalistat2 treatment (Figure 6C).

To replicate mouse observations in humans, human blood was collected from healthy subjects and treated with Lalistat2. The expression levels of PD-L1 and CSF1R in CD11c^+^ cells were gated and analyzed by flow cytometry. As demonstrated in Figure 6, D and E, and Supplemental Figure 6B, Lalistat2 treatment significantly increased the percentages of CD11c^+^ cells in the human blood and the percentages of PD-L1^+^ cells and CSF1R^+^ cells in human blood CD11c^+^ cells.

### Expression of PD-L1, CSF1R, and metabolic enzymes in immunosuppressive CD11c^+^ cells of tumor-bearing mice and cancer patients.

**Figure 7:**
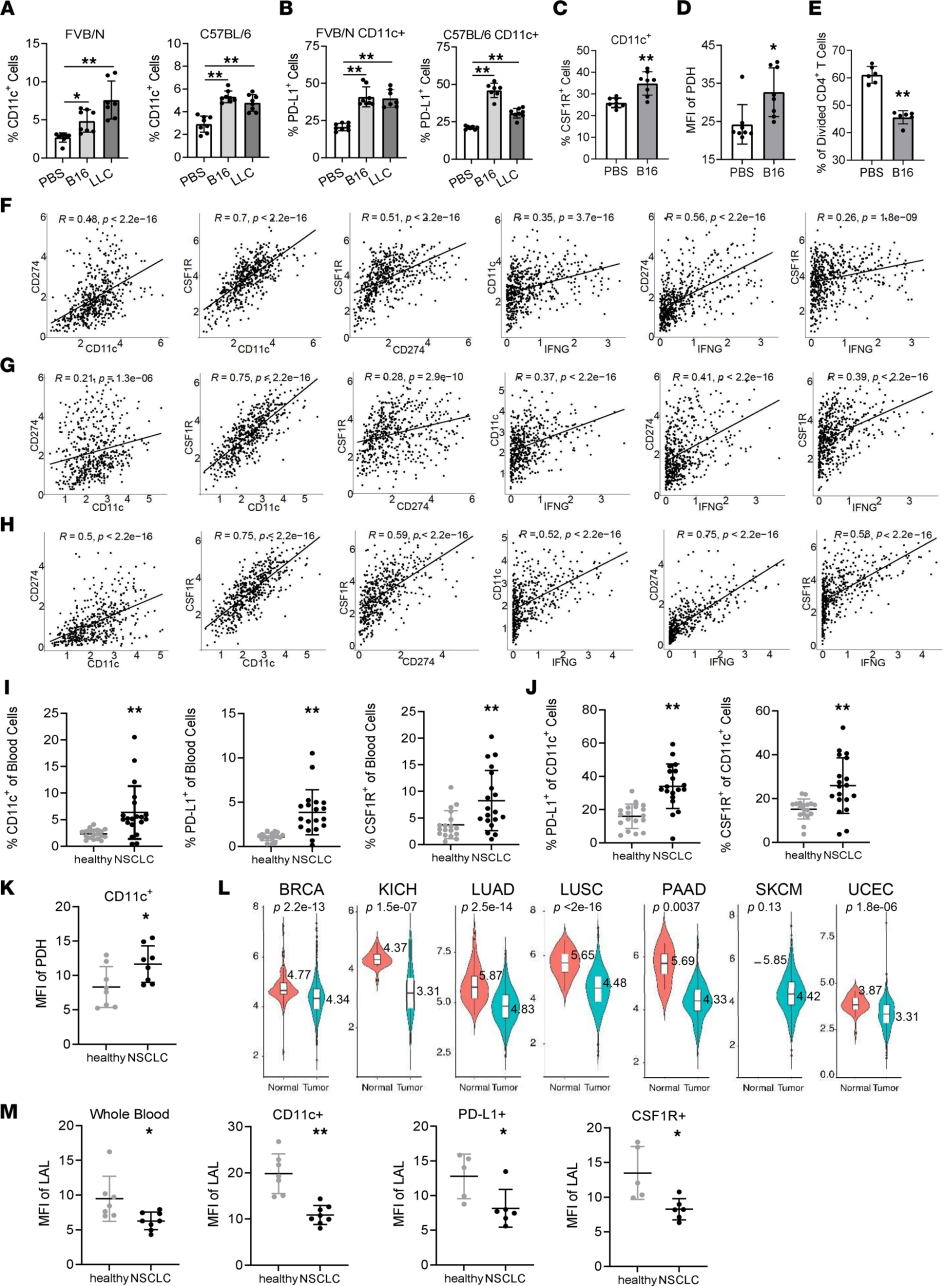
Expression of PD-L1 and CSF1R in CD11c^+^ cells of tumor-bearing mice and NSCLC patients. (**A** and **B**) Percentages of CD11c^+^ blood cells (**A**) and percentages of PD-L1^+^ cells in CD11c^+^ blood cells (**B**) of B16 melanoma or LLC cell–injected versus PBS-injected FVB/N or C57BL/6 mice. (**C** and **D**) Percentages of CSF1R^+^ cells (**C**) and MFI of PDH expression (**D**) in CD11c^+^ blood cells of B16 melanoma cell–injected versus PBS-injected FVB/N mice. (**E**) CD11c^+^ blood cells were isolated from B16 melanoma cell–injected or PBS-injected FVB/N mice, and cocultured with CFSE-labeled *Lal^+/+^* CD4^+^ T cells (at 1:1 ratio). (**F**–**H**) Pearson correlation analysis of expressions of *CD11C* and *CD274*, *CD11C* and *CSF1R*, *CD274* and *CSF1R*, *IFNG* and *CD11C*, *IFNG* and *CD274*, *IFNG* and *CSF1R* in LUAD (**F**), LUSC (**G**), and SKCM (**H**). (**I**) Statistical analysis of percentages of CD11c^+^, PD-L1^+^, and CSF1R^+^ cells in blood of patients with NSCLC versus healthy individuals. (**J**) Percentages of PD-L1^+^ and CSF1R^+^ cells in blood CD11c^+^ cells of patients with NSCLC versus healthy individuals. (**K**) MFI of PDH expression in blood CD11c^+^ cells of patients with NSCLC versus healthy individuals. (**L**) Expression of the gene *LIPA* (LAL) in violin plots from patients with BRCA, KICH, LUAD, LUSC, PAAD, SKCM, or UCEC versus healthy individuals. (**M**) MFI of LAL in whole blood, CD11c^+^, PD-L1^+^, and CSF1R^+^ cells of patients with NSCLC versus healthy individuals. Data are expressed as mean ± SD. Experiments were independently repeated, *n* = 7–8 for **A**–**D**, *n* = 6 for **E**, *n* = 585 for **F**, *n* = 550 for **G**, *n* = 472 for **H**, *n* = 18–20 for **I** and **J**, *n* = 5–8 for **K** and **M**. *P < 0.05, **P < 0.01, 1-way ANOVA for **A** and **B**, unpaired Student’s *t* test for **C**–**E**, **I**–**K**, and **M**.

The *Lal^–/–^* mouse model has a preexisting condition in favor of tumor growth. To extend the above findings to tumor-induced models (after tumor injection), B16 melanoma or LLC cells were subcutaneously injected into the flank sites of *Lal^+/+^* recipient mice in both syngeneic C57BL/6 and allogeneic FVB/N genetic backgrounds for 14 days. Blood was collected from these mice for flow cytometry analysis. As demonstrated in Figure 7A, percentages of CD11c^+^ cells in the blood were increased in tumor-bearing mice, including B16-injected FVB/N and C57BL/6 mice and LLC-injected FVB/N and C57BL/6 mice. Both the MFI and percentages of PD-L1^+^ cells in CD11c^+^ cells were upregulated in the blood of these tumor-bearing mice (Figure 7B and Supplemental Figure 7, A and B). Both the MFI and percentages of CSF1R were increased in CD11c^+^ cells of B16-injected versus PBS-injected FVB/N mice as well (Figure 7C and Supplemental Figure 7, A and C). MFI and percentages of the metabolic enzyme PDH were significantly increased in CD11c^+^ cells of B16-injected versus PBS-injected FVB/N mice (Figure 7D and Supplemental Figure 7, A and D). When cocultured with CD4^+^ T splenic cells, CD11c^+^ cells isolated from the blood of B16-injected mice suppressed T cell proliferation (Figure 7E), demonstrating that they had immunosuppressive functions similar to those of *Lal^–/–^* CD11c^+^ cells. Taken together, these results suggest that tumor-induced mice (post-tumor model) shared characteristics of *Lal^–/–^* mice (pre-tumor model) by increasing CD11c^+^ cells with immunosuppressive activity in the blood, in which PD-L1, CSF1R, and glucose metabolic enzyme were upregulated. In addition to these peripheral CD11c^+^ cells in tumor-bearing mice, Supplemental Figure 7, E and F, shows that the percentages of CD11c^+^ cells were increased in tumor tissues, accompanied by upregulated PD-L1 expression. However, the percentages of CSF1R^+^ cells in these CD11c^+^ cells were decreased. In *Lal^–/–^* mice, no increased CD11c^+^ cells were observed in tumor tissues (Supplemental Figure 7G), probably because of pool exhaustion as the myeloid compartment is already expanded in these mice. In addition, increased PD-L1 expression and decreased CSF1R level were observed in CD11c^+^ cells of tumor tissues from *Lal^–/–^* mice (Supplemental Figure 7H).

To explore whether expressions of PD-L1, CSF1R, and IFN-γ are correlated with CD11c expression in human cancer patients, data mining analyses were performed in human Lung Adenocarcinoma (LUAD), Lung Squamous Cell Carcinoma (LUSC), and Skin Cutaneous Melanoma (SKCM) samples of the TCGA database. Results showed that there were positive correlations of *CD11C* expression versus *CD274* expression, *CD11C* expression versus *CSF1R* expression, *CSF1R* expression versus *CD274* expression, *IFNG* expression versus *CD11C* expression, *IFNG* expression versus *CD274* expression, and *IFNG* expression versus *CSF1R* expression in patients with lung cancer or melanoma (Figure 7, F–H). To confirm these observations, blood was collected from healthy controls and patients with non–small cell lung cancer (NSCLC) with PD-L1–positive scores and stained with fluorescence-conjugated antibodies for flow cytometry analysis. As Figure 7I demonstrates, the percentages of CD11c^+^ cells, PD-L1^+^ cells, and CSF1R^+^ cells were all increased in the whole blood of patients with NSCLC compared with healthy controls. There were more PD-L1^+^ and CSF1R^+^ cell populations in gated CD11c^+^ cells of patients with NSCLC than in those of healthy controls (Figure 7J and Supplemental Figure 7I). In contrast, within CD11b^+^HLA-DR^–^ myeloid cells, there was relatively no change in percentages of PD-L1^+^ cells or CSF1R^+^ cells (Supplemental Figure 7, I and J). MFI and percentages of the metabolic enzyme PDH were significantly increased in CD11c^+^ cells of patients with NSCLC compared with healthy controls (Figure 7K and Supplemental Figure 7K).

Interestingly, data mining analyses of the TCGA database revealed downregulation of LAL gene expression in various human cancer forms, including Breast Cancer (BRCA), Kidney Chromophobe (KICH), LUAD, LUSC, Pancreatic Adenocarcinoma (PAAD), SKCM, and Uterine Corpus Endometrial Carcinoma (UCEC) (Figure 7L). LAL levels were further examined in blood CD11c^+^, PD-L1^+^, and CSF1R^+^ cells of patients with NSCLC by flow cytometry analysis. Importantly, Figure 7M and Supplemental Figure 7L show that the LAL levels were decreased not only in the whole blood cells but also in CD11c^+^, PD-L1^+^, and CSF1R^+^ cells of patients with NSCLC compared with healthy controls. Therefore, there is a negative correlation between LAL expression and expansion of CD11c^+^, PD-L1^+^, and CSF1R^+^ cells in patients with NSCLC, similar to that observed in the *Lal^–/–^* model and tumor-bearing mice.

## Discussion

Antitumor immune suppression is a complicated process, which involves both T cell and myeloid cell components in the ecosystem of tumor microenvironment. Among all immune therapies, the PD-L1/PD-1 checkpoint pathway has so far been proven to be a valuable therapeutic target to eradicate malignancies. Engagement of PD-1 by PD-L1 alters the activity of T cells in many ways, including inhibiting T cell proliferation, survival, cytokine production, and other effector functions. In our study, PD-L1 was subjected to neutral lipid regulation that was controlled by LAL. In *Lal^–/–^* mice, increased PD-L1 expression was observed in whole blood (Figure 1A). Since the *Lal^–/–^* mouse model provides a favorable environment for tumor growth and metastasis (17), it would be intriguing to determine whether PD-L1 plays a role in this environment and reveal which immune components contribute to the increase of PD-L1 expression. It is unlikely that increased PD-L1 expression came from the T cell component, because *Lal^–/–^* mice have impaired development and proliferation of T cells (20), which results in extremely low levels of T cells in most immune organs. In contrast, *Lal^–/–^* mice have robust proliferation of myeloid cells that is initiated in the early stages of myelopoiesis in the bone marrow (16, 18, 30). Therefore, the search for the increased PD-L1 expression was mainly focused on the myeloid lineage of *Lal^–/–^* mice.

Flow cytometry analysis using various myeloid surface markers revealed that PD-L1 expression was only upregulated in CD11c^+^ myeloid cells of *Lal^–/–^* mice (Figure 1, B and C). Functionally, isolated *Lal^–/–^* CD11c^+^ blood cells possessed strong T cell suppression and stimulation of tumor cells (Figure 2), which are 2 hallmarks of *Lal^–/–^* MDSCs (16–18). Based on these similarities and standards, *Lal^–/–^* CD11c^+^ cells are referred to as CD11c^+^ MDSCs in order to distinguish them from traditional CD11b^+^Ly6G^+^ MDSCs in *Lal^–/–^* mice. Unlike MDSCs from tumor-bearing mice that are divided into “monocytic” and “granulocytic” MDSCs (34–36), almost all traditional *Lal^–/–^* MDSCs are Ly6G^+^Ly6C^+^ (18). CD11c is typically considered to be a marker of conventional DCs (11–14), although it has been reported to be expressed on monocytes, granulocytes, macrophages, and a subset of B cells, T cells, and natural killer cells (37–41). It appears that CD11c^+^ cells are a highly plastic population with diverse functions in various disease models. These diverse populations were also observed in the *Lal^+/+^* and *Lal^–/–^* blood, although most CD11c^+^ cells possess either neutrophil (cluster 0236) or monocyte (cluster 158) features (Supplemental Table 1).

It is important to distinguish similarities and differences between *Lal^–/–^* CD11b^+^Ly6G^+^ MDSCs and *Lal^–/–^* CD11c^+^ MDSCs at the cellular and molecular levels. In fat catabolism, triglycerides are hydrolyzed to break into fatty acids and glycerol by LAL. Fatty acids are further broken down through the process of β-oxidation that results in acetyl-CoA, which can be used in the tricarboxylic acid (TCA) cycle in mitochondria. In the absence of the regular supply of fatty acids during LAL deficiency, cells inevitably use substitutive energy consumption pathways to fuel oxidative phosphorylation (OXPHOS). In *Lal^–/–^* CD11b^+^Ly6G^+^ MDSCs, Affymetrix GeneChip microarray and QIAGEN Ingenuity Pathway Analysis identified the upregulation of many enzymes that are involved in the glycolytic pathway and the TCA cycle. In addition, gene expressions of lactate dehydrogenases, enzymes in the pentose phosphate pathway (energy conservation for biosynthetic purposes), and glycogen synthesis (storage form of glucose and metabolic energy) were upregulated in *Lal^–/–^* CD11b^+^Ly6G^+^ MDSCs (42). In the present study, using Seahorse analysis for extracellular acidification rate and oxygen consumption rate measurements, both glycolytic metabolism and OXPHOS in mitochondrial respiration were significantly increased in *Lal^–/–^* CD11c^+^ MDSCs, accompanied by higher ATP production (Figure 4, E and F). scRNA-Seq and t-SNE clustering approaches showed that genes involved in glycolysis and the TCA cycle were upregulated in *Lal^–/–^* CD11c^+^ MDSCs (Figure 4, A and B; and Supplemental Figure 3, A and B). These observations were confirmed by flow cytometry analysis in which protein expression levels of several key glucose downstream metabolic enzymes (PDH, G6PD, LDH) and GLUD were increased in *Lal^–/–^* CD11c^+^ MDSCs (Figure 4G). During the process of glycolysis, glucose is broken down into pyruvate, which moves into the mitochondria. PDH plays a role in converting pyruvate into acetyl-CoA by decarboxylation to enter the TCA cycle in mitochondria. G6PD and LDH control the pentose phosphate pathway and anaerobic glycolysis, respectively. GLUD participates in the glutamine pathway. Taken together, these results suggest a metabolic switch to overuse glucose and amino acids as the energy source in both *Lal^–/–^* CD11b^+^Ly6G^+^ MDSCs and *Lal^–/–^* CD11c^+^ MDSCs. Importantly, inhibition of PDH by the pharmacological inhibitor CPI-613 not only reduced the population of *Lal^–/–^* CD11c^+^ MDSCs and their PD-L1 expression (Figure 4H) but also reversed their capabilities of T cell suppression and tumor growth stimulation (Figure 4, I and J). Mitochondria help to control various metabolic decision points that determine immune cell functions (43). In *Lal^–/–^* CD11b^+^Ly6G^+^ MDSCs, ROS were overproduced, accompanied by damaged mitochondrial function as a penalty of overusing glucose and amino acids (30). A similar finding was observed in *Lal^–/–^* CD11c^+^ MDSCs. There were major distinctions between gene expression patterns and trajectory lineage differentiation of the ROS pathway in cluster 158 and cluster 0236 of *Lal^–/–^* CD11c^+^ MDSCs compared with *Lal^+/+^* CD11c^+^ cells (Figure 4D and Supplemental Figure 3, D and E), which was associated with high-level ROS production (Figure 4C). The high level of ROS allows for the stimulation of cell proliferation, induction of genetic instability, and evasion from senescence (44). Accordingly, gene expression of glutathione reductase (*Gsr*), a critical enzyme in redox regulation, was upregulated in *Lal^–/–^* CD11c^+^ MDSCs (Supplemental Figure 3F). Clearly, both *Lal^–/–^* CD11b^+^Ly6G^+^ MDSCs and *Lal^–/–^* CD11c^+^ MDSCs share a specialized metabolism that differs from that of their normal counterparts. Metabolic switch and plasticity in the tumor environment compromise immune-mediated tumor destruction, and offer an opportunity to selectively target functions of immune cells for enhancing effective immunotherapy (25, 27).

However, there are significant differences between *Lal^–/–^* CD11b^+^Ly6G^+^ MDSCs and *Lal^–/–^* CD11c^+^ MDSCs. At least 2 biomarkers (PD-L1 and CSF1R) can be used to distinguish *Lal^–/–^* CD11b^+^Ly6G^+^ MDSCs and *Lal^–/–^* CD11c^+^ MDSCs. First, increased PD-L1 expression was only observed in *Lal^–/–^* CD11c^+^ MDSCs, not in *Lal^–/–^* CD11b^+^Ly6G^+^ MDSCs (Figure 1). Pretreatment with anti–PD-L1 antibody reversed *Lal^–/–^* CD11c^+^ MDSC suppressive activity of T cells (Figure 2C) and stimulation of tumor growth (Figure 2E). Blockade of PD-L1 on DCs has been reported to enhance T cell activation and cytokine production (10). In murine tumor-infiltrating DCs, blockade of PD-L1 also resulted in a better capability of stimulating T cell activation, contributing to a more potent ability to inhibit tumor growth in mice (9, 45). Second, CSF1R expression was increased in cluster 158 of *Lal^–/–^* CD11c^+^ MDSCs by scRNA-Seq and flow cytometry analyses but not in *Lal^–/–^* CD11b^+^Ly6G^+^ MDSCs (Figure 5, A and B, and Supplemental Figure 5B). Similarly to PD-L1 blockade, pretreatment with anti-CSF1R antibody reversed *Lal^–/–^* CD11c^+^ MDSCs’ suppressive activity on T cells (Figure 5C) and stimulation of tumor growth (Figure 5D). These observations in the genetically ablated system were confirmed in the murine HD1A myeloid cell line by the pharmacological LAL inhibitor Lalistat2 (Figure 6, A–C). More importantly, Lalistat2 treatment induced PD-L1 and CSF1R expression in human blood CD11c^+^ myeloid cells (Figure 6, D and E). Therefore, increased PD-L1 expression and CSF1R expression are the unique features of *Lal^–/–^* CD11c^+^ MDSCs that can be used to distinguish them from *Lal^–/–^* CD11b^+^Ly6G^+^ MDSCs. On the other hand, there was a report showing exclusive overexpression of fatty acid transport protein 2 (FATP2) that was controlled by GM-CSF through the activation of the STAT5 transcription factor in PMN-MDSCs. Selective inhibition of FATP2 abrogated the activity of PMN-MDSCs and substantially delayed tumor progression (46).

In addition to the increased levels of PD-L1 and CSF1R, *Lal^–/–^* CD11c^+^ MDSCs possess many other unique features. The scRNA-Seq approach identified that CD11c^+^ MDSCs could be divided into 2 major cellular clusters in t-SNE clustering analysis (cluster 158 and cluster 0236). Compared with *Lal^+/+^* CD11c^+^ cells, *Lal^–/–^* CD11c^+^ MDSCs showed increased cellular number in cluster 158 but decreased cellular number in cluster 0236 (Figure 3A). Considering that *Lal^–/–^* CD11c^+^ MDSCs possessed immunosuppressive and tumor stimulatory functions instead of DCs’ traditional antigen-presenting immune stimulatory function, these 2 clusters may represent 2 subpopulations with distinctive functions. It has been reported that DCs switch from an immunostimulatory activation state driving antitumor immunity in early-stage tumors to an immunosuppressive activation state at later stages (47). The secretion of immunosuppressive factors by cancer cells has been proposed to be implicated in the control of DC differentiation, maturation, and function (48). Similarly here, cluster 158 in *Lal^–/–^* CD11c^+^ MDSCs may contribute to their immunosuppressive and tumor stimulatory functions. Indeed, the level of the gene *Cd274* (PD-L1) was significantly increased in cluster 158 and slightly decreased in cluster 0236 of *Lal^–/–^* CD11c^+^ MDSCs compared with *Lal^+/+^* CD11c^+^ cells (Figure 3B). As demonstrated in Figure 2, *Lal^–/–^* CD11c^+^ MDSCs exerted their immunosuppressive and tumor stimulatory functions by upregulating PD-L1 expression. This was controlled by CSF1R, which has a gene expression pattern similar to that of PD-L1 in cluster 158 and cluster 0236 (Figure 5A). This CSF1R/PD-L1 axis in *Lal^–/–^* CD11c^+^ MDSCs was, at least in part, responsible for their ability to suppress T cells and stimulate tumor growth (Figure 5, C and D). As demonstrated in Figure 3, C–E, and Supplemental Tables 2–7, many other genes and pathways may also contribute to functional switch of CD11c^+^ cells in the *Lal^–/–^* protumor microenvironment. This opens a door for future investigation. For example, flow cytometry analysis showed upregulation of IFN-γ, MCP-1, GM-CSF, and IL-10 proteins in *Lal^–/–^* CD11c^+^ cells versus *Lal^+/+^* CD11c^+^ cells (Figure 1E). Among them, IFN-γ has been reported to regulate PD-L1 expression (21). Indeed, the percentage of PD-L1^+^ cells was decreased in *Lal^–/–^* CD11c^+^ cells with IFN-γ–neutralizing antibody treatment (Supplemental Figure 1F). There were more cells positive for *Ifng* (gene name of IFN-γ) in cluster 158 of *Lal^–/–^* versus *Lal^+/+^* CD11c^+^ cells (Supplemental Figure 2). Blocking CSF1R did not significantly change the IFN-γ level in *Lal^–/–^* CD11c^+^ cells (Supplemental Figure 5D), suggesting that PD-L1 is regulated by at least 2 independent pathways in these cells.

Although *Lal^–/–^* mice are a pre-tumor model, the findings observed in this model can be extended to tumor-bearing models. In both B16 melanoma–bearing and LLC tumor–bearing mice, not only were the percentages of CD11c^+^ cells in the blood increased, but expression of PD-L1, CSF1R, and PDH in CD11c^+^ cells was also upregulated (Figure 7, A–D). Functionally, these CD11c^+^ cells from the B16 melanoma–induced model suppressed T cell proliferation (Figure 7E). Besides, the percentages of CD11c^+^ cells and the percentages of PD-L1^+^ cells in CD11c^+^ cells were also increased in the tumor tissues of these tumor-bearing mice (Supplemental Figure 7, E and F). Thus, tumor-bearing mice shared similar characteristics with *Lal^–/–^* mice, and CD11c^+^ MDSC upregulation is a common feature in both pre-tumor models (*Lal^–/–^*) and post-tumor (tumor-induced) models.

Importantly, in the correlation analyses in human cancer patients by data mining of TCGA database, there were positive correlations among *CD11C*, *CD274*, *CSF1R*, and *IFNG* expression in lung adenocarcinoma, lung squamous carcinoma, and melanoma (Figure 7, F–H). In addition, human NSCLC patients with PD-L1–positive scores confirmed the increased percentages of CD11c^+^ cells and increased PD-L1, CSF1R, and PDH expression in gated CD11c^+^ cells from blood (Figure 7, I–K). In contrast, increase of PD-L1 or CSF1R expression was not observed in CD11b^+^HLA-DR^–^ myeloid cells (representing PMN-MDSCs) (Supplemental Figure 7, I and J), confirming that the increased expression of PD-L1 and CSF1R was only associated with CD11c^+^ cells, and less associated with PMN-MDSCs, in patients with NSCLC. Therefore, CD11c^+^ MDSC upregulation is common in the tumor environment of both tumor-bearing animal models and human patients with cancer. This holds great potential for the design of CD11c^+^ cell–based immunotherapy to enhance the efficacy of T cell–based checkpoint immunotherapy for cancer treatment. Recently, there was a report showing that PD-L1 was universally upregulated in tumor-derived LAMP3^+^ conventional DCs in almost all human cancer types (49).

Based on the present studies and previous publications, CD11c^+^ cells seem to possess dual functions depending on genetic defects and existing microenvironments, and can be divided into 2 subgroups: (a) an antitumor subgroup with APC/T cell–stimulating/tumor-suppressive functions, and (b) a protumor subgroup with T cell–suppressive/tumor-stimulating functions. The LAL/CSF1R/PD-L1 axis is a major driver in the transition between these 2 subgroups through neutral lipid to glycolysis metabolic reprogramming.

Last but not least, LIPA expression was downregulated in multiple forms of human cancers by data mining of the TCGA database (Figure 7L). Flow cytometry analysis further showed downregulation of LAL in CD11c^+^, PD-L1^+^, and CSF1R^+^ cells of blood samples from NSCLC patients (Figure 7M). Therefore, observations made from both mouse models (*Lal^–/–^* and tumor-bearing) and human cancer patients support a notion that LAL plays a suppressive role in these oncogenic processes and can be used for cancer immunotherapy purposes. LAL (sebelipase alfa [Kanuma]) can be used to treat patients with cancer as immunotherapy to decrease protumor immune cells in clinical settings. It is conceivable that LAL negatively promotes CSF1R and PD-L1 at the transcriptional level. LAL is well known for its role to activate nuclear receptors (e.g., PPARs) by generating hormone ligands. How CSF1R and PD-L1 genes are downregulated by nuclear receptors at the transcriptional level warrants further investigation. We noticed that several Fos/Jun subunits of AP-1 transcription factor were co-upregulated in cluster 158 but downregulated in cluster 0236 (Figure 3D). It is intriguing to see the connections between them and regulation of CSF1R and PD-L1 genes as well.

## Methods

### Animals and cell lines.

Wild-type (*Lal^+/+^*) and *Lal^−/−^* mice of the FVB/N and C57BL/6 background were bred in-house (50, 51). Humanized *c-fms*-rtTA/(TetO)_7_-CMV-hLAL (*c-fms*–Tg/KO) mice of the FVB/N background are a previously generated triple-transgenic mouse model with myeloid-specific doxycycline-inducible expression of wild-type human LAL (hLAL) in *Lal^−/−^* mice under the control of the *c-fms* promoter (18, 52). Both male and female mice aged 3–4 months were used, and all the mice had been backcrossed for more than 10 generations.

The murine B16 melanoma cell line and Lewis lung carcinoma (LLC) cell line (ATCC) were cultured in DMEM supplemented with 10% FBS (Gibco) in a 37°C incubator with 5% CO_2_. The HD1A myeloid cell line was previously established in our laboratory (33) and cultured in RPMI 1640 (Gibco) supplemented with 10% FBS in a 33°C incubator with 8% CO_2_. To inhibit LAL activity, HD1A cells were treated with 10 μM, 50 μM, 100 μM, or 200 μM LAL inhibitor Lalistat2 (Cayman) for 72 hours (31, 32). As a control, HD1A cells were treated with DMSO.

### Human blood samples.

The human blood samples of normal subjects were obtained from Indiana Biobank, and NSCLC (stage III–IV) patients were accessed through Simon Comprehensive Cancer Center of Indiana University School of Medicine after signing a consent form. Patients with NSCLC were selected by screening with PD-L1 Tumor Proportion Score ≥ 10%. Both healthy participants and patients with NSCLC included male and female, White and African American. To inhibit LAL activity, human blood cells from healthy participants had the red blood cells (RBCs) removed with lysis buffer (BioLegend), washed with PBS by centrifugation at 240*g* for 5 minutes at room temperature, and incubated with 10 μM LAL inhibitor Lalistat2 for 24 hours. As a control, human blood cells were incubated with DMSO.

### Flow cytometry analysis.

*Lal^+/+^* and *Lal^–/–^* mice were euthanized, and blood was immediately collected from the posterior vena cava. The blood was then treated with RBC lysis buffer to remove RBCs and washed with PBS. For analysis of PD-L1 and CSF1R expression in immune cell subtypes, cells were stained with APC-conjugated anti-CSF1R antibody (AFS98, catalog 17-1152-82), APC–eFluor 780–conjugated anti-Ly6G antibody (1A8-Ly6g, catalog 47-9668-82), APC–eFluor 780–conjugated anti-F4/80 antibody (BM8, catalog 47-4801-82), FITC-conjugated anti-CD11b antibody (M1/70, catalog 11-0112-82), FITC-conjugated anti-MHCII antibody (M5/114.15.2, catalog 11-5321-82), and PE-conjugated anti-CD11c antibody (N418, catalog 12-0114-82) (eBioscience); FITC-conjugated anti-Ly6C antibody (HK1.4, catalog 128006, BioLegend); and PE-Cy7–conjugated anti–PD-L1 antibody (10F.9G2, catalog 124314, BioLegend) at 4°C for 15 minutes. Cells were washed with PBS, then were ready for flow cytometry analysis.

For analyses of cytokine and metabolic enzyme levels in CD11c^+^ cells, isolated cells were first stained with PE-conjugated or APC-conjugated (N418, catalog 17-0114-82) anti-CD11c antibody at 4°C for 15 minutes. After being fixed and permeabilized using BD Cytofix/Cytoperm Fixation/Permeabilization Kit (BD Biosciences), cells were incubated with fluorescence-conjugated antibodies against intracellular molecules including APC-conjugated anti–IFN-γ antibody (XMG1.2, catalog 17-7311-82), APC-conjugated anti–IL-2 antibody (JES6-5H4, catalog 17-7021-82), APC-conjugated anti–IL-10 antibody (JES5-16E3, catalog 17-7101-82), FITC-conjugated anti–GM-CSF antibody (MP1-22E9, catalog 17-7331-82), FITC-conjugated anti–IL-1β antibody (NJTEN3, catalog 17-7114-80), and PE-conjugated anti–MCP-1 antibody (2H5, catalog 12-7096-82) (eBioscience), and non-fluorescence-conjugated antibodies against metabolic molecules including G6PD (D5D2, catalog 12263S), LDH (C28H7, catalog 3558S), PDH (catalog 2784S), and GLUD (D9F7P, catalog 12793S) (Cell Signaling Technology), at 4°C overnight. On the next day, for non-fluorescence-conjugated antibodies, cells were washed and stained with Alexa Fluor 647–conjugated anti-rabbit IgG antibody (catalog 4414S) (Cell Signaling Technology) at 4°C for 30 minutes, then washed for flow cytometry analysis.

For flow cytometry analysis of human blood samples, human blood cells had the RBCs removed, washed with PBS, and stained with APC-conjugated anti-CSF1R antibody, APC–eFluor 780–conjugated anti-CD11c antibody (3.9, catalog 47-0116-42), PE-conjugated anti–PD-L1 antibody (MIH1, catalog 12-5983-42), PE-Cy5–conjugated anti-CD11b antibody (ICRF44, catalog 15-0118-42), and PE-Cy7–conjugated anti–HLA-DR antibody (L243, catalog 25-9952-42) (eBioscience) at 4°C for 15 minutes. Cells were then washed with PBS, fixed with 1% paraformaldehyde, and analyzed by flow cytometry analysis. To analyze LAL levels, cells were further fixed and permeabilized using BD Cytofix/Cytoperm Fixation/Permeabilization Kit and incubated with non-fluorescence-conjugated anti-LAL antibody (53) at 4°C overnight. On the next day, cells were washed and stained with FITC-conjugated anti-rabbit IgG antibody at 4°C for 30 minutes, then washed for flow cytometry analysis.

For flow cytometry analysis, ≥50,000 cells were acquired and scored using an LSRII machine (mouse samples) or Fortessa (human samples) (BD Biosciences). Data were processed using BD CellQuest Pro software (version 19.f3fcb) and FlowJo (version 10.6.1) (BD Biosciences).

### Isolation of blood CD11c^+^ cells.

Blood was collected from the posterior vena cava of mice. After removal of RBCs and PBS washing, cells were first incubated with biotin-conjugated anti-CD11c antibody (N418, catalog 130-125-219, Miltenyi Biotec) at 4°C for 30 minutes. After washing with PBS, cells were incubated with anti-biotin microbeads at 4°C for another 30 minutes. Subsequently, cells were subjected to magnetic bead sorting according to the manufacturer’s instructions (Miltenyi Biotec). For blocking antibody treatment, freshly isolated CD11c^+^ cells were pretreated with IgG or anti–PD-L1 (5 μg/mL) (10F.9G2, catalog 124302, BioLegend) or anti-CSF1R antibodies (5 μg/mL) (AFS98, catalog 14-1152-82, eBioscience) or anti–IFN-γ (5 μg/mL) (37895, catalog MAB485-100, R&D Systems) at 4°C for 1 hour, and then cocultured with CD4^+^ T cells or coinjected with B16 melanoma cells for further analysis. For CPI-613 treatment, freshly isolated CD11c^+^ cells were pretreated with DMSO or 10 μM CPI-613 at 37°C for 30 minutes, then cocultured with CD4^+^ T cells or coinjected with B16 melanoma cells for further analysis.

### T cell proliferation assay.

CD4^+^ T cells were isolated from the spleen and labeled with CFSE as previously described (20). CFSE-labeled CD4^+^ T cells were then cocultured with isolated CD11c^+^ cells in 96-well plates precoated with anti-CD3 mAb (2 μg/mL) (145-2C11, catalog 553057) and anti-CD28 mAb (5 μg/mL) (37.51, catalog 553295) (BD Biosciences) at 37°C, 5% CO_2_, for 4 days. The ratio of CD11c^+^ cells to CD4^+^ T cells was 1:1. Proliferation of CD4^+^ T cells was evaluated as CFSE dilution by flow cytometry analysis.

### Subcutaneous injection of tumor cells into Lal^+/+^ mice.

To study the effects of CD11c^+^ cells on tumor growth, isolated CD11c^+^ cells (2 × 10^5^) with or without pretreatment were mixed with B16 melanoma cells (2 × 10^5^), and the cell mixture was injected subcutaneously at the flank region of *Lal^+/+^* recipient mice. The tumor growth was monitored twice a week. The tumor volume (mm^3^) was estimated by measurement of the maximal length and width of a tumor and calculated using the formula (length × width^2^)/2.

For tumor-bearing mouse experiments, wild-type (*Lal^+/+^*) mice of the FVB/N or C57BL/6 background were injected with 1 × 10^6^ B16 melanoma or LLC cells at flank sites on 2 sides. Fourteen days later, the mice were sacrificed, and the blood was collected for flow cytometry analysis. To analyze the levels of CD11c^+^, PD-L1^+^, or CSF1R^+^ cells in tumors, tumor tissues from B16 melanoma–injected *Lal^+/+^* or *Lal^–/–^* FVB/N mice were harvested, digested for single-cell preparation, and stained for flow cytometry analysis.

### Single-cell RNA sequencing and data analysis.

CD11c^+^ cells were sorted from the blood of *Lal^+/+^* and *Lal^−/−^* mice as described above. Briefly, blood cells from *Lal^+/+^* or *Lal^−/−^* mice had the RBCs removed and were washed with PBS twice and then incubated with anti-CD11c microbeads for magnetic bead sorting. Cells obtained after sorting were washed twice with PBS to remove debris and resuspended in PBS. To obtain equal cell numbers, cells sorted from 10 *Lal^+/+^* mice and 4 *Lal^−/−^* mice were pooled together and mixed well. The number and viability of CD11c^+^ cells were 1100 cells/μL and >95%, respectively. Immediately after sorting, CD11c^+^ single cells were run on the 10X Chromium (10X Genomics) and then through library preparation by the Center for Medical Genomics at Indiana University School of Medicine following the recommended protocol for the Chromium Single Cell 3′ Reagent Kit. Libraries were run on the NovaSeq S1 for Illumina sequencing.

CellRanger 3.0.2 (http://support.10xgenomics.com/) was used to process the raw sequence data generated. CellRanger used bcl2fastq (https://support.illumina.com/) to demultiplex raw base sequence calls generated from the sequencer into sample-specific FASTQ files. The FASTQ files were then aligned to the mouse reference genome mm10 with RNA-Seq aligner STAR. The aligned reads were traced back to individual cells, and the gene expression level of individual genes was quantified based on the number of unique molecular identifiers (UMIs) detected in each cell. The filtered gene-cell barcode matrices generated by CellRanger were used for further analysis. Cells with unique gene counts over 8000 or having greater than 10% mitochondrial genes were filtered out. The gene expression was normalized by total expression of the cell, multiplied by a scaling factor of 10,000, and log_2_-transformed. Two samples were integrated using FindIntegrationAnchors in Seurat package (54). After scaling of the integrated data, the first 13 principal components from principal component analysis were used to cluster the cells by a shared nearest neighbor modularity optimization–based clustering algorithm (55). Clusters were annotated to cell subtypes using SingleR package (56). Visualization was performed with Seurat package. Differential gene expression analysis was analyzed using Wilcoxon’s rank sum test with Seurat package. Trajectory analysis was performed by monocle 2.16.0 (57–59) on ROS response genes (from the Mouse Genome Informatics database, http://www.informatics.jax.org/go/term/GO:0000302) using plot_cell_trajectory function after dimensionality reduction with DDRTree algorithm. Data were deposited in the NCBI’s Gene Expression Omnibus database (GEO GSE206837).

### Pearson correlation analysis of gene expression of CD11c, PD-L1, CSF1R, and IFN-γ from the TCGA database.

Cancer Genome Atlas Lung Adenocarcinoma (TCGA-LUAD) (60) (585 samples), Lung Squamous Cell Carcinoma (TCGA-LUSC) (61) (550 samples), and Skin Cutaneous Melanoma (TCGA-SKCM) (472 samples) data were retrieved from the UCSC Xena platform (62), which is wholly or partly based on data generated by the TCGA Research Network (https://www.cancer.gov/tcga). Normalized gene expressions of *CD274*, *CD11C*, *CSF1R*, and *IFNG* were compared with each other in the tumor samples. Pearson correlation and its *P* value were calculated. Expression comparisons of the gene *LIPA* (LAL) in Breast Cancer (BRCA), Kidney Chromophobe (KICH), LUAD, LUSC, Pancreatic Adenocarcinoma (PAAD), SKCM, or Uterine Corpus Endometrial Carcinoma (UCEC) patients versus healthy control individuals were also mined from TCGA, including various demographic variables.

### ROS measurement.

The ROS level in CD11c^+^ cells was measured by flow cytometry as previously described (63). White blood cells from *Lal^+/+^* and *Lal^–/–^* mice were collected as described above and stained with PE-conjugated anti-CD11c antibody and 2 μmol/L 2′,7′-dichlorofluorescein diacetate (Invitrogen) at 37°C for 30 minutes. After PBS washing, the ROS level in CD11c^+^ cells was analyzed using an LSRII machine.

### Extracellular acidification rate and oxygen consumption rate (Seahorse) assays.

Extracellular acidification rate (ECAR) and oxygen consumption rate (OCR) measurements were performed using a Seahorse Bioscience XF-96 instrument as described previously (64, 65). On the day before the experiment, the sensor cartridge was hydrated overnight using the calibration buffer supplied by the manufacturer (Agilent). On the day of the experiment, CD11c^+^ cells were freshly isolated as described above, and washed with calibration buffer twice. For ECAR measurement, CD11c^+^ cells were incubated with glucose-free Seahorse XF base medium supplemented with 2 mM glutamine for 1 hour at 37°C in a CO_2_-free incubator. The injection ports of the sensors were filled with 20–25 μL of treatment or vehicle in buffer. The sensor was then placed into the XF-96 instrument and calibrated. After calibration, the calibration fluid plate was replaced with the cell plate. The measurement cycle consisted of a 2-minute mix, a 1-minute wait, and a 2-minute measurement. Four basal rate measurements were followed by sequential addition of glucose (100 mM), oligomycin (10 μM), and 2-deoxyglucose (500 mM) prepared in glucose-free Seahorse XF base medium. Each injection was followed by 4 measurement cycles.

For OCR measurement, CD11c^+^ cells were incubated with glucose-free Seahorse XF base medium supplemented with 1 mM pyruvate, 2 mM glutamine, and 10 mM glucose for 1 hour at 37°C in a CO_2_-free incubator. The following procedure was similar to ECAR measurement, except that 4 basal rate measurements were followed by sequential addition of oligomycin (100 μM), carbonyl cyanide-4 (trifluoromethoxy)phenylhydrazone (FCCP; 100 μM), and rotenone/antimycin A (50 μM) prepared in glucose-free Seahorse XF base medium. The consumption rates were calculated from the continuous average slope of the decreased O_2_ using a compartmentalization model (66). For CD11c^+^ cells from different genotypes, the rates from 8 wells were used.

### LAL activity measurement.

The LAL enzymatic activity was determined using 4-methylumbelliferyl oleate (4-MUO; MilliporeSigma) as substrate as described previously (51). Proteins were extracted from HD1A myeloid cells, which had been treated with Lalistat2 or DMSO for 72 hours. Protein concentrations were determined by Pierce BCA protein assay kit (Thermo Fisher Scientific). Eighteen micrograms of protein was added to 0.567 mM substrate solution (0.567 mM 4-MUO, 0.15 M sodium acetate/0.01% Tween-80, pH 5.5, 1% [vol/vol] Triton X-100). Reaction was incubated at 37°C for 30 minutes, and then terminated by addition of 100 μL of 0.75 M Tris, pH 8.0. A standard curve was prepared ranging from 0 to 100 nM 4-methylumbelliferone (MilliporeSigma). Fluorescence was measured on a Gemini XPS plate reader (Molecular Devices) at 355 nm excitation and 460 nm emission. Data were analyzed using the SoftMax program. Assays were linear within the time frame of these assays, and less than 10% of substrate was cleaved. One unit is 1 mmol of 4-MUO cleaved per minute under standard assay conditions.

### Statistics.

Data are expressed as mean ± SD. Differences between 2 treatment groups were compared by 2-tailed Student’s *t* test. When more than 2 groups were compared, 1-way ANOVA with post hoc Newman-Keuls multiple-comparison test was used. When the data were entered into a grouped table with subcolumns, 2-way ANOVA with multiple-comparison test was used. A *P* value less than 0.05 was considered statistically significant. All analyses were performed with GraphPad Prism 8.4.1.

### Study approval.

All scientific protocols involving the use of animals were approved by the Institutional Animal Care and Use Committee of Indiana University School of Medicine and followed guidelines established by the Panel on Euthanasia of the American Veterinary Medical Association. Animals were housed under Institutional Animal Care and Use Committee–approved conditions in a secured animal facility at Indiana University School of Medicine. All protocols involving the use of human blood were approved by the Institutional Biosafety Committee of Indiana University School of Medicine, and written informed consent was received prior to participation.

## Author contributions

TZ designed and performed experiments, analyzed and interpreted the data, and wrote the manuscript. JW and SL analyzed and interpreted the scRNA-Seq data. XD performed flow cytometry. EMJ maintained mouse colonies and performed genotyping. NHH provided the human NSCLC blood samples. KS and CKS were responsible for Seahorse analysis. HD and CY designed experiments, analyzed and interpreted the data, and wrote the manuscript.

## Supplementary Material

Supplemental data

## Version 1 08/02/2022

In-Press Preview

## Version 2 09/08/2022

Electronic publication
