# Supplementary material for: Lysosomal acid lipase, CSF1R, and PD-L1 determine functions of CD11c+ myeloid-derived suppressor cells
Source: JCI Insight. 2022 Sep 8;7(17):e156623. doi: 10.1172/jci.insight.156623 (PMC9536279; doi:10.1172/jci.insight.156623)
Supplement: Supplemental data [file jciinsight-7-156623-s038.pdf]

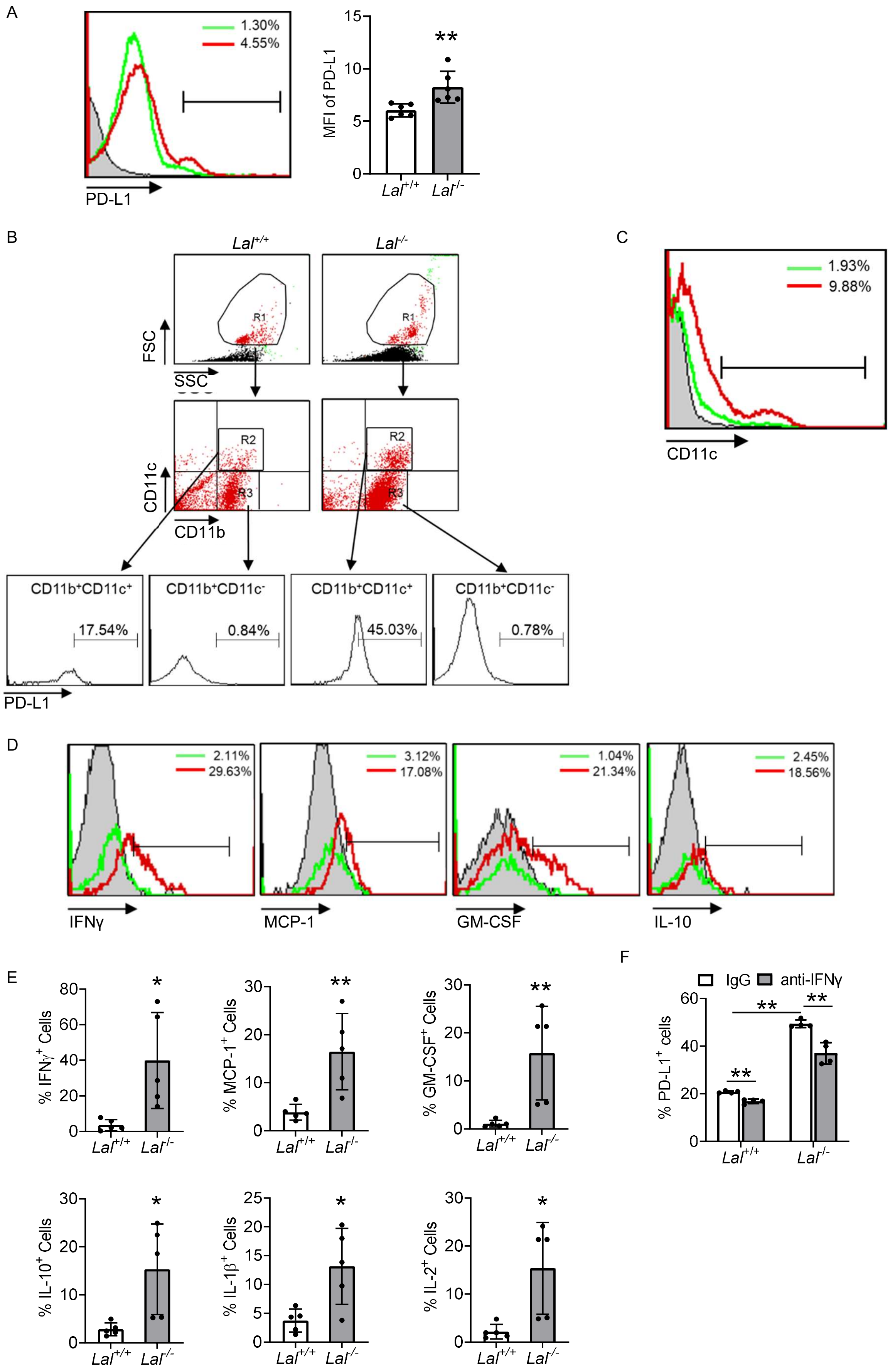
A
1.30%
4.55%
PD-L1
MFI of PD-L1
**
Lal+/+
Lal-/-
B
FSC
SSC
R1
CD11c
CD11b
R2
R3
CD11b+CD11c+
17.54%
CD11b+CD11c-
0.84%
45.03%
0.78%
C
1.93%
9.88%
CD11c
D
2.11%
29.63%
IFNγ
3.12%
17.08%
MCP-1
1.04%
21.34%
GM-CSF
2.45%
18.56%
IL-10
E
% IFNγ+ Cells
% MCP-1+ Cells
% GM-CSF+ Cells
% IL-10+ Cells
% IL-1β+ Cells
% IL-2+ Cells
F
IgG
anti-IFNγ
% PD-L1+ cells

**Supplemental Figure 1. Gating strategies of PD-L1$^+$ and CD11c$^+$ cells and percentages of cytokine-expressing cells in CD11c$^+$ cells.** **A)** White blood cells harvested from *Lal*$^{+/+}$ and *Lal*$^{-/-}$ mice were stained with fluorescence-conjugated antibodies against PD-L1, CD11c, MHCⅡ, F4/80, CD11b, Ly6C, and Ly6G, and analyzed by flow cytometry. Left: A representative gating strategy of PD-L1$^+$ cells in the blood. Right: MFI of PD-L1 expression in the blood of *Lal*$^{-/-}$ vs. *Lal*$^{+/+}$ mice by flow cytometry analysis. **B)** A representative gating strategy of PD-L1$^+$ cells in CD11b$^+$CD11c$^-$ and CD11b$^+$CD11c$^+$ cells. **C)** A representative gating strategy of CD11c$^+$ cells in the blood. **D)** Expression levels of cytokines in *Lal*$^{-/-}$ vs. *Lal*$^{+/+}$ CD11c$^+$ cells were analyzed by flow cytometry. Gating strategies of IFNγ, MCP-1, GM-CSF, and IL-10 in CD11c$^+$ cells. **E)** The percentages of IFNγ$^+$, MCP-1$^+$, GM-CSF$^+$, IL-10$^+$, IL-1β$^+$, and IL-2$^+$ cells in *Lal*$^{-/-}$ vs. *Lal*$^{+/+}$ CD11c$^+$ cells. **F)** The percentage of PD-L1$^+$ cells in blood CD11c$^+$ cells after treated with 5 μg/mL IFNγ neutralizing antibody for 2 days. For (A), (C) and (D), the gray-shaded area is isotype control. The green and red lines represent the signals of *Lal*$^{+/+}$ and *Lal*$^{-/-}$ cells, respectively. The bracket shows the gating of "positives". Data are expressed as mean ± SD; Experiments were independently repeated, n=6 for A-C, n=5 for D-E, n=4 for F. *p<0.05, **p<0.01, unpaired Student's *t* test for A and E, 2-way ANOVA for F.

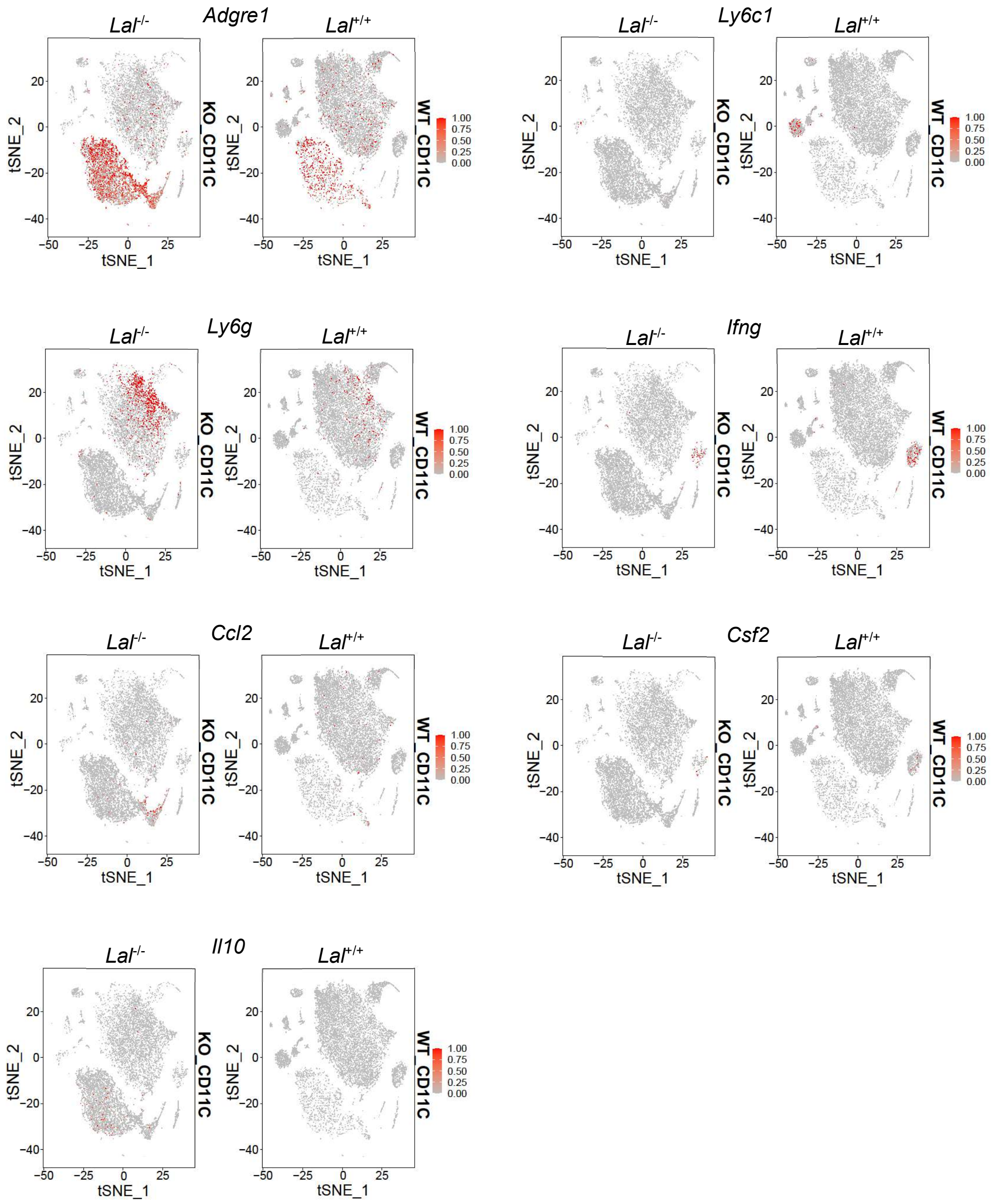


**Supplemental Figure 2. Gene expressions across cell clusters in tSNE plots of CD11c$^{+}$ cells.** Gene expressions of F4/80 (*Adgre1*), Ly6C (*Ly6c1*), Ly6G (*Ly6g*), IFNγ (*Ifng*), MCP-1 (*Ccl2*), GM-CSF (*Csf2*), and IL-10 (*Il10*) across cell clusters in tSNE plots of CD11c$^{+}$ cells from *Lal*$^{-/-}$ vs. *Lal*$^{+/+}$ mice.

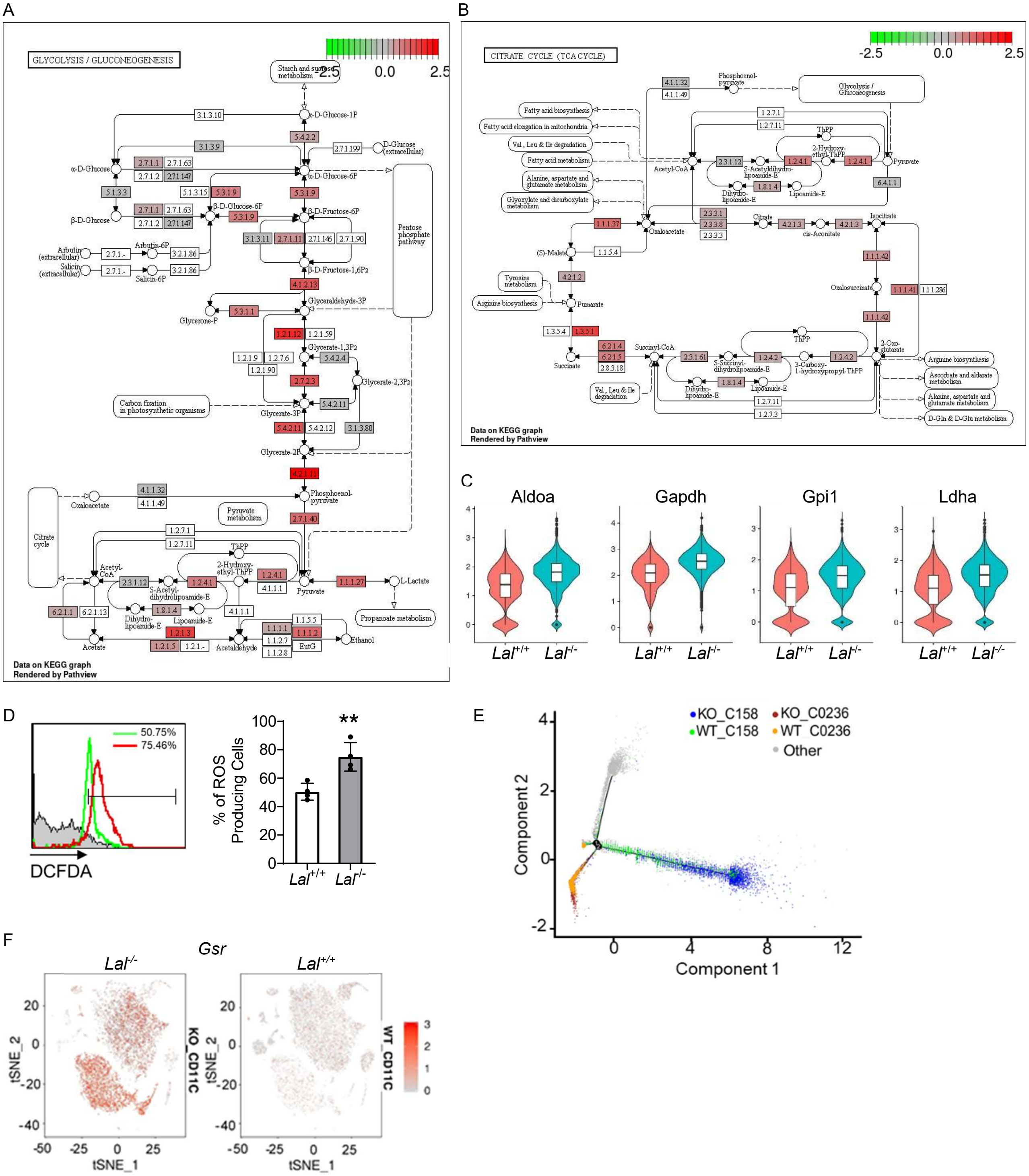

A
GLYCOLYSIS / GLUCONEOGENESIS
-2.5 0.0 2.5
Data on KEGG graph
Rendered by Pathview
B
CITRATE CYCLE (TCA CYCLE)
-2.5 0.0 2.5
Data on KEGG graph
Rendered by Pathview
C
Aldoa
Gapdh
Gpi1
Ldha
Lal+/+
Lal-/-
D
50.75%
75.46%
DCFDA
% of ROS Producing Cells
**
Lal+/+
Lal-/-
E
KO_C158
KO_C0236
WT_C158
WT_C0236
Other
Component 2
Component 1
F
Gsr
Lal-/-
Lal+/+
KO_CD11C
WT_CD11C
tSNE_2
tSNE_1

**Supplemental Figure 3. Expression of metabolic enzymes and ROS in *Lal*$^{-/-}$ CD11c$^{+}$ cells.** White blood cells were isolated from *Lal*$^{+/+}$ and *Lal*$^{-/-}$ mice for flow cytometry and scRNA sequencing. **A)** Differential expression of genes involved in glycolysis/gluconeogenesis in *Lal*$^{-/-}$ vs. *Lal*$^{+/+}$ CD11c$^{+}$ cells (red boxes represent increased expression of enzymes). **B)** Differential expression of genes involved in citrate cycle in *Lal*$^{-/-}$ vs. *Lal*$^{+/+}$ CD11c$^{+}$ cells (red boxes represent increased expression of enzymes). **C)** Expression of glycolysis-related gene *Aldoa, Gapdh, Gpil* and *Ldha* across cluster 158 in violin plots of CD11c$^{+}$ cells from *Lal*$^{-/-}$ vs. *Lal*$^{+/+}$ mice. **D)** Left: Gating strategies of ROS-producing cells in CD11c$^{+}$ cells. The gray-shaded area is isotype control. The green and red lines represent the signals of *Lal*$^{+/+}$ and *Lal*$^{-/-}$ CD11c$^{+}$ cells, respectively. The bracket shows the gating of "positives". Right: The percentage of ROS-producing cells in *Lal*$^{-/-}$ vs. *Lal*$^{+/+}$ CD11c$^{+}$ cells by flow cytometry analysis. **E)** Group distribution in a two-dimensional independent component space generated by Monocle's pseudotime ordering of the cells. Solid black line indicates the main diameter path of the Minimum Spanning Tree (MST) and acts as the backbone of the trajectory. **F)** *Gsr* expression across cell clusters in tSNE plots of CD11c$^{+}$ cells from *Lal*$^{-/-}$ vs. *Lal*$^{+/+}$ mice by scRNA sequencing. Data are expressed as mean ± SD; Experiments were independently repeated, n=4 for D. $^{**}p<0.01$, unpaired Student's *t* test.

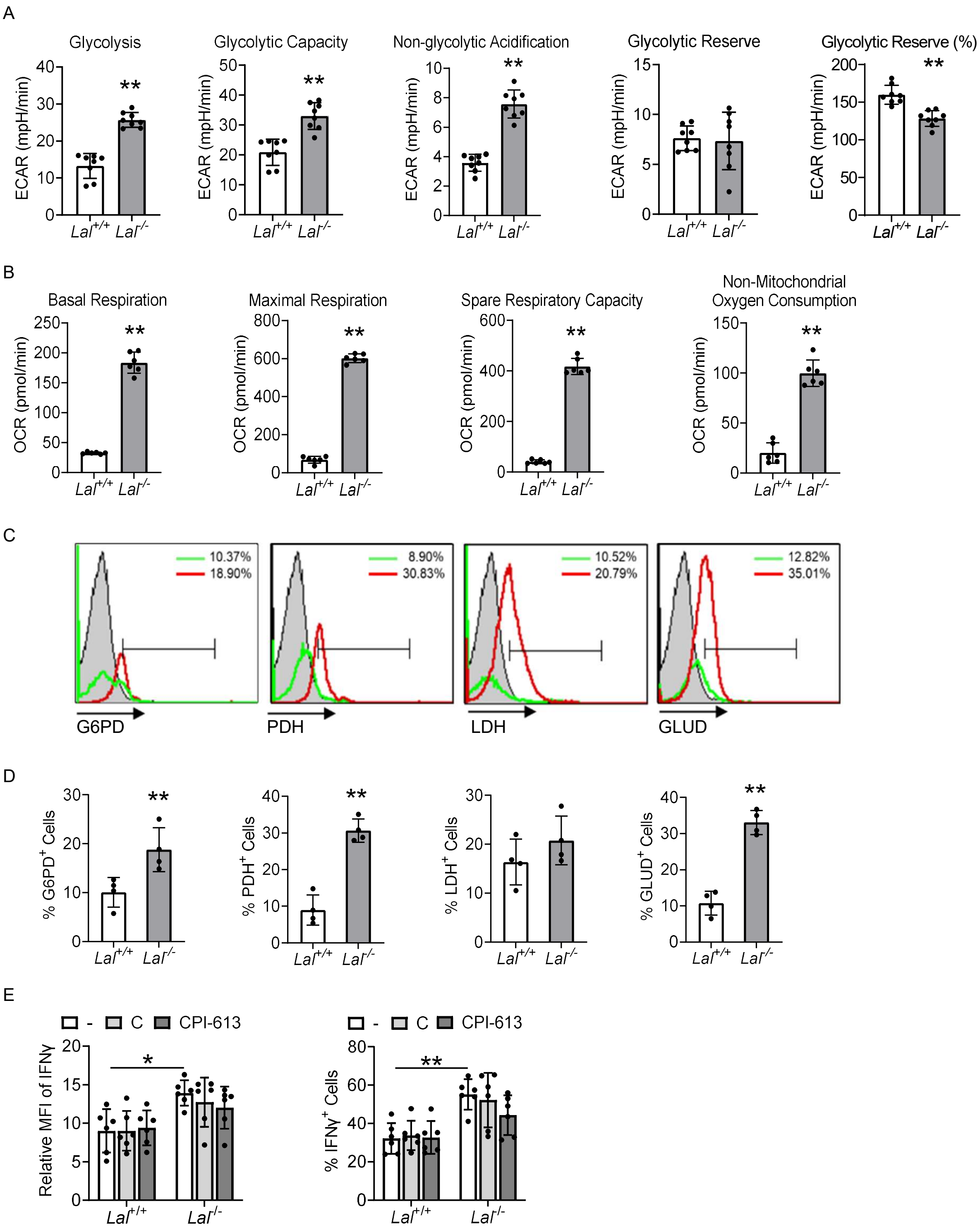
A
Glycolysis
Glycolytic Capacity
Non-glycolytic Acidification
Glycolytic Reserve
Glycolytic Reserve (%)
ECAR (mpH/min)
**
Lal+/+ Lal-/-
B
Basal Respiration
Maximal Respiration
Spare Respiratory Capacity
Non-Mitochondrial Oxygen Consumption
OCR (pmol/min)
C
10.37%
18.90%
8.90%
30.83%
10.52%
20.79%
12.82%
35.01%
G6PD
PDH
LDH
GLUD
D
% G6PD+ Cells
% PDH+ Cells
% LDH+ Cells
% GLUD+ Cells
E
- C CPI-613
Relative MFI of IFNγ
% IFNγ+ Cells
*
**

**Supplemental Figure 4. Expression of metabolic enzymes in *Lal*$^{-/-}$ CD11c$^{+}$ cells. A)** Glycolysis, glycolytic capacity, non-glycolytic acidification, glycolytic reserve and glycolytic reserve (%) measured from Seahorse XF96. **B)** Basal and maximal respiration, spare respiratory capacity, and non-mitochondrial oxygen consumption measured from Seahorse XF96. **C)** Gating strategies of G6PD, PDH, LDH, and GLUD in CD11c$^{+}$ cells by flow cytometry. The gray-shaded areas are isotype controls. The green and red lines represent the signals of *Lal*$^{+/+}$ and *Lal*$^{-/-}$ CD11c$^{+}$ cells, respectively. The bracket shows the gating of "positives". **D)** The percentages of G6PD$^{+}$, PDH$^{+}$, LDH$^{+}$ and GLUD$^{+}$ cells in *Lal*$^{-/-}$ vs. *Lal*$^{+/+}$ CD11c$^{+}$ cells by flow cytometry analysis. **E)** Relative MFI of IFNγ and percentages of IFNγ$^{+}$ cells in CD11c$^{+}$ cells after CPI-613 treatment by flow cytometry analysis. Data are expressed as mean ± SD; Experiments were independently repeated, n=8 for A, n=6 for B and E, n=4 for D. **$p<0.01$, unpaired Student's *t* test for A, B and D, 2-way ANOVA for E.

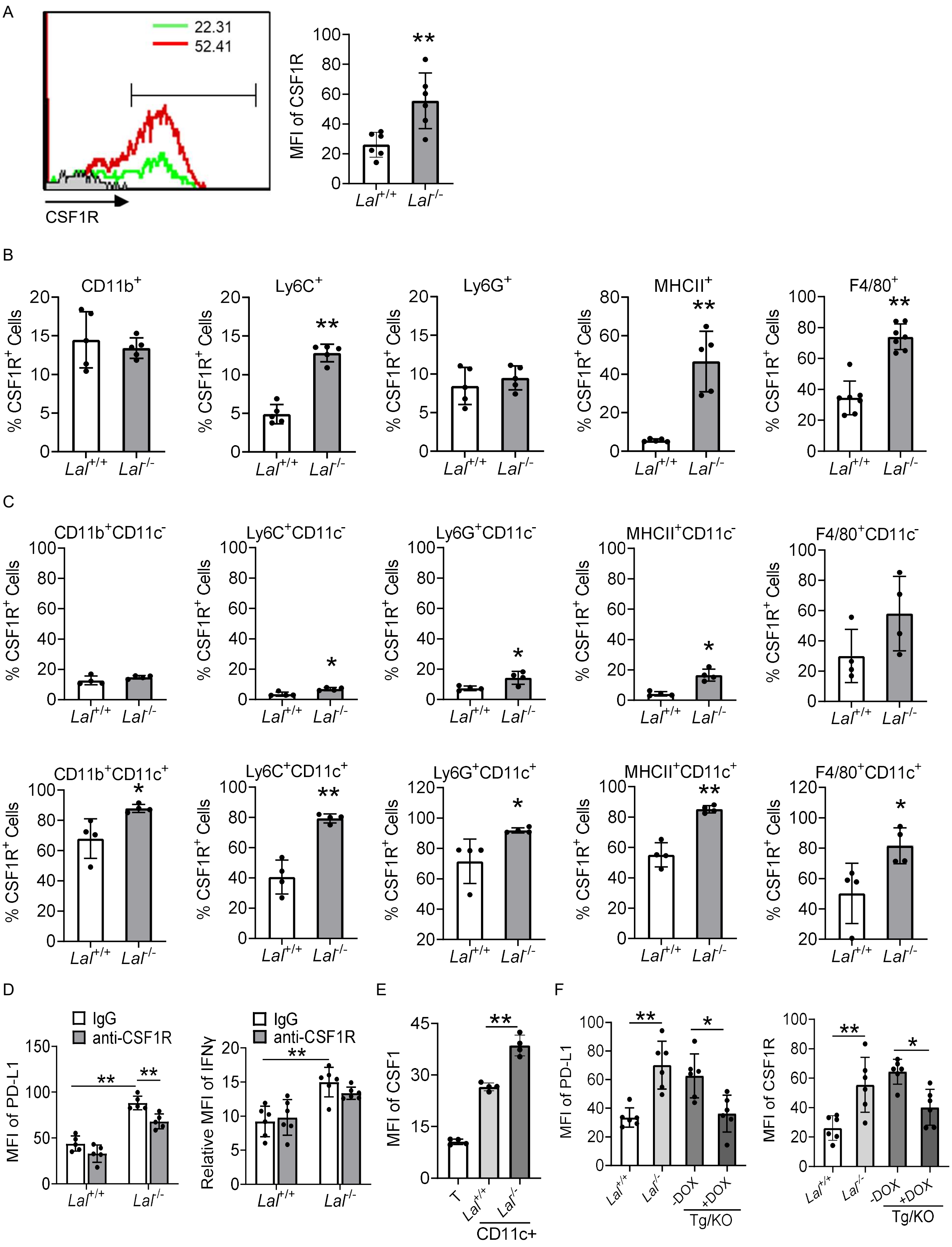
A
22.31
52.41
CSF1R
MFI of CSF1R
Lal+/+
Lal-/-
**
B
CD11b+
Ly6C+
Ly6G+
MHCII+
F4/80+
% CSF1R+ Cells
C
CD11b+CD11c-
Ly6C+CD11c-
Ly6G+CD11c-
MHCII+CD11c-
F4/80+CD11c-
CD11b+CD11c+
Ly6C+CD11c+
Ly6G+CD11c+
MHCII+CD11c+
F4/80+CD11c+
D
IgG
anti-CSF1R
MFI of PD-L1
Relative MFI of IFNγ
E
MFI of CSF1
T
CD11c+
F
-DOX
+DOX
Tg/KO

**Supplemental Figure 5. CSF1R expression in *Lal*$^{-/-}$ CD11c$^{+}$ cells.** **A)** MFI of CSF1R in blood CD11c$^{+}$ cells of *Lal*$^{-/-}$ vs. *Lal*$^{+/+}$ mice by flow cytometry analysis. Left: Gating strategies of CSF1R in CD11c$^{+}$ cells. The gray-shaded area is isotype control. The green and red lines represent the signals of *Lal*$^{+/+}$ and *Lal*$^{-/-}$ CD11c$^{+}$ cells, respectively. The bracket shows the gating of "positives". **B)** CSF1R expression in blood CD11b$^{+}$, Ly6C$^{+}$, Ly6G$^{+}$, CD11b$^{+}$, MHCII$^{+}$, F4/80$^{+}$ cells of *Lal*$^{-/-}$ vs. *Lal*$^{+/+}$ mice by flow cytometry analysis. **C)** CSF1R expression in CD11c$^{-}$ and CD11c$^{+}$ double-gated myeloid cells by flow cytometry analysis. **D)** MFI of PD-L1 and IFNγ expression in blood CD11c$^{+}$ cells after anti-CSF1R antibody treatment (5 μg/mL) by flow cytometry analysis. **E)** MFI of CSF1 expression in spleen CD4$^{+}$ T cells and blood CD11c$^{+}$ cells of *Lal*$^{+/+}$ and *Lal*$^{-/-}$ mice by flow cytometry analysis. **F)** MFI of CSF1R and PD-L1 expression in blood CD11c$^{+}$ cells of *Lal*$^{+/+}$, *Lal*$^{-/-}$, untreated (-DOX), and DOX-treated (+DOX) Tg/KO mice by flow cytometry analysis. Data are expressed as mean ± SD; Experiments were independently repeated, n=6 for A and F, n=5 for B, n=5-6 for D, and n=4 for C and E. $^{*}p<0.05$, $^{**}p<0.01$, unpaired Student's *t* test for A-C, 2-way ANOVA for D, 1-way ANOVA for E and F.

A

B

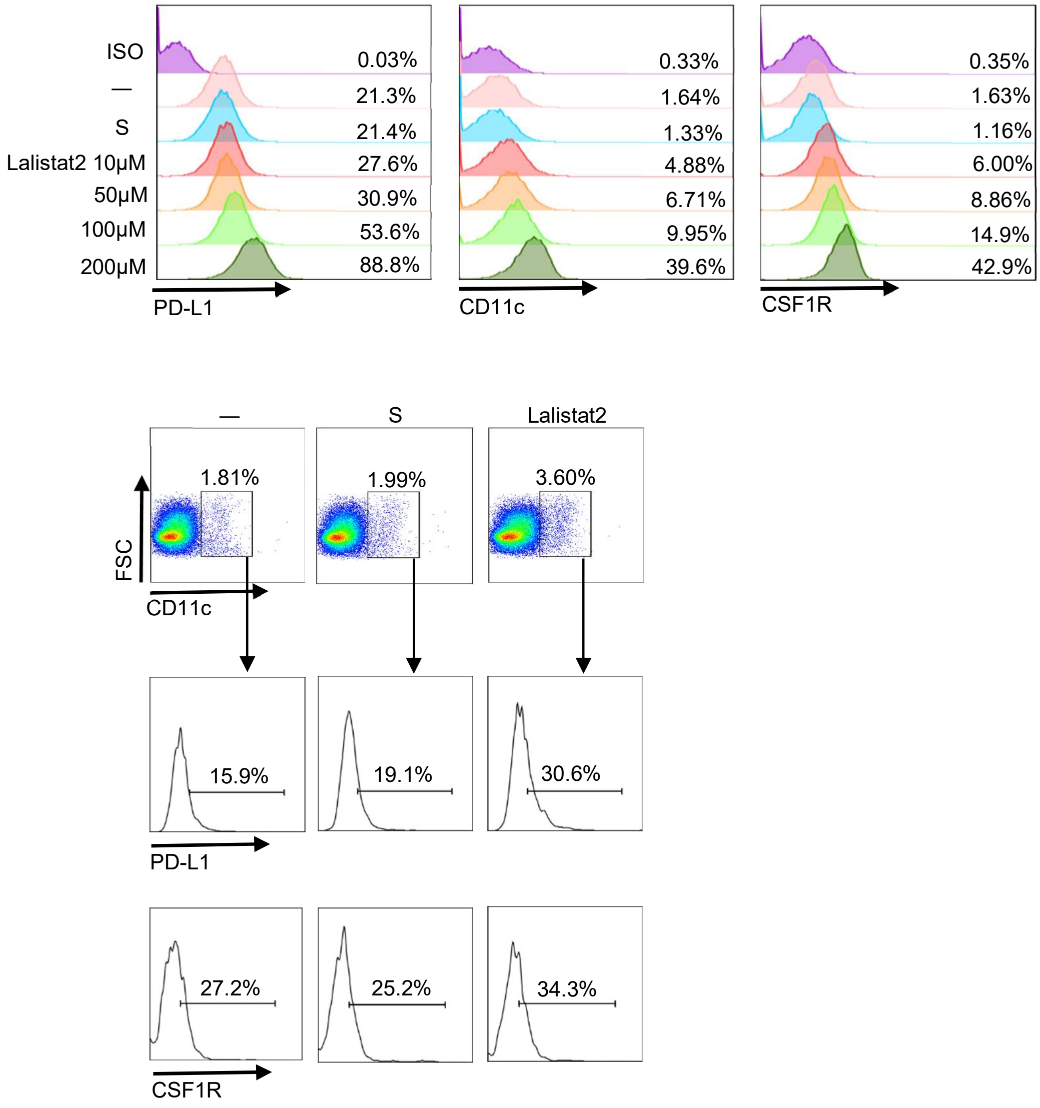


**Supplemental Figure 6. Expression of PD-L1 and CSF1R in mouse myeloid cells and human blood CD11c$^+$ cells after Lalistat2 treatment. A)** A representative gating strategy of PD-L1$^+$, CD11c$^+$ and CSF1R$^+$ cells in HD1A myeloid cells treated with Lalistat2 or DMSO. **B)** A representative gating strategy of PD-L1$^+$ and CSF1R$^+$ cells in CD11c$^+$ cells of human blood cells treated with Lalistat2 at 10 μM or DMSO (S).

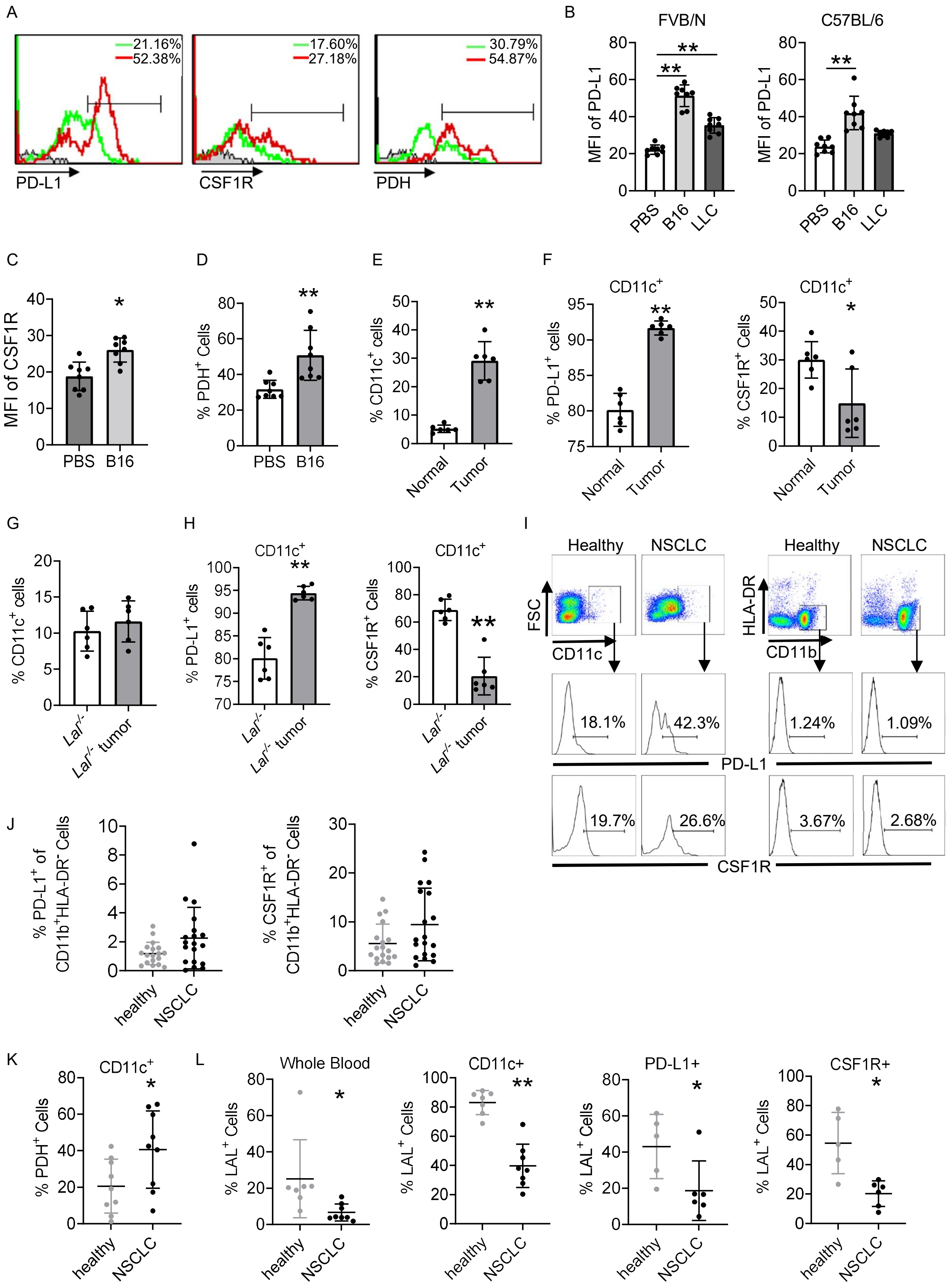

A
21.16%
52.38%
PD-L1
17.60%
27.18%
CSF1R
30.79%
54.87%
PDH
B
FVB/N
C57BL/6
MFI of PD-L1
PBS
B16
LLC
C
MFI of CSF1R
D
% PDH+ Cells
E
% CD11c+ cells
Normal
Tumor
F
CD11c+
% PD-L1+ cells
% CSF1R+ Cells
G
% CD11c+ cells
Lal-/-
Lal-/- tumor
H
I
Healthy
NSCLC
FSC
CD11c
HLA-DR
CD11b
18.1%
42.3%
1.24%
1.09%
PD-L1
19.7%
26.6%
3.67%
2.68%
CSF1R
J
% PD-L1+ of CD11b+HLA-DR- Cells
% CSF1R+ of CD11b+HLA-DR- Cells
healthy
NSCLC
K
L
Whole Blood
CD11c+
PD-L1+
CSF1R+
% LAL+ Cells

**Supplemental Figure 7. Expressions of PD-L1 and CSF1R in CD11c$^+$ cells of tumor bearing mice and NSCLC patients. A)** Representative gating strategies of PD-L1, CSF1R, and PDH in CD11c$^+$ cells. The gray-shaded areas are isotype controls. The green and red lines represent the signals of CD11c$^+$ cells from PBS-injected and B16 melanoma cell-injected FVB/N *Lal*$^{+/+}$ mice, respectively. The bracket shows the gating of "positives". **B)** MFI of PD-L1 in blood CD11c$^+$ cells of B16 melanoma or LLC cell-injected vs. PBS-injected FVB/N or C57BL/6 *Lal*$^{+/+}$ mice by flow cytometry. **C)** MFI of CSF1R in blood CD11c$^+$ of B16 melanoma cell-injected vs. PBS-injected FVB/N *Lal*$^{+/+}$ mice. **D)** The percentage of PDH$^+$ cells in blood CD11c$^+$ cells of B16 melanoma cell-injected vs. PBS-injected FVB/N *Lal*$^{+/+}$ mice. **E)** The percentage of CD11c$^+$ cells in tumor tissues and **F)** the percentages of PD-L1$^+$ and CSF1R$^+$ cells in CD11c$^+$ cells of tumors tissues from B16 melanoma cell-injected FVB/N *Lal*$^{+/+}$ mice. **G)** The percentage of CD11c$^+$ cells in tumor tissues and **H)** the percentages of PD-L1$^+$ and CSF1R$^+$ cells in CD11c$^+$ cells of tumors tissues from B16 melanoma cell-injected FVB/N *Lal*$^{-/-}$ mice. **I)** A representative gating strategy of PD-L1$^+$ and CSF1R$^+$ cells in CD11c$^+$ cells and CD11b$^+$HLA-DR$^-$ cells of NSCLC patients vs. healthy individuals. **J)** Statistical analysis of percentages of PD-L1$^+$ and CSF1R$^+$ cells in blood CD11b$^+$HLA-DR$^-$ of NSCLC patients vs. healthy individuals. **K)** The percentage of PDH$^+$ cells in blood CD11c$^+$ cells of NSCLC patients vs. healthy individuals. **L)** The percentages of LAL$^+$ cells in whole blood, CD11c$^+$, PD-L1$^+$, and CSF1R$^+$ cells of NSCLC patients vs. healthy individuals. Data are expressed as mean ± SD; Experiments were independently repeated, n=8 for B-D, n=6 for E-H, n=17-19 for J, n=5-9 for K and L. *p<0.05, **p<0.01, 1-way ANOVA for B, unpaired Student's *t* test for C-H, J-L.

**Supplemental Table 1. Identification and gene expression of *Lal*$^{-/-}$ vs. *Lal*$^{+/+}$ CD11c$^{+}$ cell clusters by scRNA-seq.**

| Cluster | Labels | Ratios of CD11c$^{+}$ Cells | | # of CD11c$^{+}$ Cells | |
|---|---|---|---|---|---|
| | | *Lal*$^{+/+}$ | *Lal*$^{-/-}$ | *Lal*$^{+/+}$ | *Lal*$^{-/-}$ |
| 0 | Neutrophils (GN) | 27.88 | 17.9 | 3009 | 1827 |
| 1 | Monocytes (MO.6C-IIINT) | 8.77 | 38.1 | 946 | 3888 |
| 2 | Neutrophils (GN) | 15.81 | 18.76 | 1706 | 1914 |
| 3 | Neutrophils (GN) | 15.39 | 2.75 | 1661 | 281 |
| 4 | T cells (T.CD4TESTCJ) | 7.28 | 0.44 | 786 | 45 |
| 5 | Monocytes (MO.6C-IIINT) | 0.86 | 6.7 | 93 | 684 |
| 6 | Neutrophils (GN) | 6.41 | 0.64 | 692 | 65 |
| 7 | NK cells (NK.DAP10-) | 5.23 | 1.39 | 564 | 142 |
| 8 | Monocytes (MO.6C+II-) | 0.32 | 3.97 | 35 | 405 |
| 9 | Neutrophils (GN) | 2.21 | 1.89 | 239 | 193 |
| 10 | DC (DC.PDC.8-) | 1.78 | 1.57 | 192 | 160 |
| 11 | B cells (B.T3) | 2.09 | 0.56 | 226 | 57 |
| 12 | Neutrophils (GN) | 0.92 | 1.73 | 99 | 177 |
| 13 | Monocytes (MO.6C-II+) | 0.97 | 0.86 | 105 | 88 |
| 14 | T cells (T.CD4TESTCJ) | 1.34 | 0.44 | 145 | 45 |
| 15 | T cells (T.8EFF.OT1.48HR.LISOVA) | 0.48 | 0.98 | 52 | 100 |
| 16 | Monocytes (MO.6C-IIINT) | 0.73 | 0.56 | 79 | 57 |
| 17 | Neutrophils (GN) | 0.57 | 0.24 | 61 | 24 |
| 18 | Basophils (BA) | 0.68 | 0.02 | 73 | 2 |
| 19 | Monocytes (MO.6C-IIINT) | 0.06 | 0.27 | 7 | 28 |
| 20 | Macrophages (MF.II-480HI) | 0.04 | 0.21 | 4 | 21 |
| 21 | B cells (B1A) | 0.17 | 0.02 | 18 | 2 |

## Supplemental Table 2. Up-regulated genes in *Lal*$^{-/-}$ CD11c$^{+}$ cells (top 50)

| Genes | Symbol | LogFC | # of cells | | Expression | |
|---|---|---|---|---|---|---|
| | | | *Lal*$^{+/+}$ | *Lal*$^{-/-}$ | *Lal*$^{+/+}$ | *Lal*$^{-/-}$ |
| lectin, galactose binding, soluble 3 | Lgals3 | 1.85 | 4759 | 9697 | 2.03 | 3.24 |
| glutathione reductase | Gsr | 1.84 | 2156 | 9397 | 1.48 | 2.33 |
| chitinase-like 3 | Chil3 | 1.76 | 249 | 4290 | 1.54 | 2.04 |
| WAP four-disulfide core domain 17 | Wfdc17 | 1.68 | 3036 | 6554 | 2.39 | 2.76 |
| interferon induced transmembrane protein 1 | Ifitm1 | 1.68 | 5727 | 6260 | 2.31 | 3.23 |
| hypoxanthine guanine phosphoribosyl transferase | Hprt | 1.45 | 6 | 6473 | 0.9 | 1.68 |
| placenta-specific 8 | Plac8 | 1.39 | 3067 | 7706 | 2.28 | 2.81 |
| cytochrome b-245, beta polypeptide | Cybb | 1.32 | 2439 | 6277 | 2.26 | 2.80 |
| neutrophilic granule protein | Ngp | 1.27 | 142 | 1031 | 1.73 | 2.30 |
| membrane-spanning 4-domains, subfamily A, member 6C | Ms4a6c | 1.24 | 1466 | 5439 | 1.66 | 2.18 |
| lipocalin 2 | Lcn2 | 1.18 | 2023 | 3508 | 2.23 | 2.55 |
| peroxiredoxin 1 | Prdx1 | 1.17 | 3011 | 6202 | 1.58 | 2.14 |
| cathepsin B | Ctsb | 1.17 | 4382 | 7619 | 2.00 | 2.64 |
| serum amyloid A 3 | Saa3 | 1.16 | 41 | 1054 | 1.45 | 1.94 |
| transmembrane protein 14C | Tmem14c | 1.16 | 1124 | 7349 | 1.21 | 1.56 |
| interferon induced transmembrane protein 6 | Ifitm6 | 1.10 | 1874 | 6571 | 1.85 | 1.90 |
| predicted pseudogene 10320 | Gm10320 | 1.08 | 28 | 5990 | 1.05 | 1.35 |
| WAP four-disulfide core domain 21 | Wfdc21 | 1.06 | 3740 | 4615 | 2.37 | 2.92 |
| lysozyme 2 | Lyz2 | 1.05 | 6580 | 9304 | 3.07 | 3.96 |
| cathepsin S | Ctss | 1.03 | 2896 | 6761 | 2.08 | 2.52 |
| interferon induced transmembrane protein 3 | Ifitm3 | 0.99 | 4598 | 8862 | 2.38 | 2.93 |
| S100 calcium binding protein A4 | S100a4 | 0.99 | 1619 | 5455 | 2.29 | 2.36 |
| guanine nucleotide binding protein (G protein), gamma transducing activity polypeptide 2 | Gngt2 | 0.98 | 4665 | 7237 | 2.14 | 2.67 |
| coagulation factor X | F10 | 0.95 | 305 | 4593 | 1.11 | 1.42 |
| superoxide dismutase 2, mitochondrial | Sod2 | 0.93 | 1867 | 5651 | 1.23 | 1.58 |
| cathepsin C | Ctsc | 0.93 | 1723 | 5174 | 1.22 | 1.63 |
| adhesion G protein-coupled receptor E4 | Adgre4 | 0.89 | 1140 | 4911 | 1.80 | 1.83 |
| insulin-like growth factor binding protein 6 | Igfbp6 | 0.89 | 663 | 3017 | 1.81 | 1.80 |
| sphingomyelin phosphodiesterase, acid-like 3A | Smpdl3a | 0.86 | 3011 | 6862 | 1.75 | 2.03 |
| histocompatibility 2, class II antigen E beta | H2-Eb1 | 0.85 | 313 | 1262 | 1.02 | 1.95 |
| CD302 antigen | Cd302 | 0.83 | 577 | 4922 | 1.16 | 1.37 |
| colony stimulating factor 1 receptor | Csf1r | 0.83 | 3292 | 6659 | 1.92 | 2.16 |
| annexin A5 | Anxa5 | 0.83 | 1223 | 5196 | 1.15 | 1.46 |
| sphingomyelin phosphodiesterase, acid-like 3B | Smpdl3b | 0.81 | 670 | 4328 | 1.25 | 1.45 |
| uridine phosphorylase 1 | Upp1 | 0.80 | 304 | 3749 | 1.68 | 1.42 |
| Fc receptor, IgE, high affinity I, gamma polypeptide | Fcer1g | 0.79 | 8849 | 9915 | 2.80 | 3.47 |
| cathepsin Z | Ctsz | 0.79 | 3911 | 7110 | 1.77 | 2.08 |
| peroxiredoxin 5 | Prdx5 | 0.78 | 7448 | 9201 | 2.06 | 2.56 |
| mitochondrial ribosomal protein L52 | Mrpl52 | 0.77 | 1825 | 5555 | 1.17 | 1.47 |
| angiotensin I converting enzyme (peptidyl-dipeptidase A) 1 | Ace | 0.75 | 1157 | 4615 | 2.03 | 1.85 |
| apolipoprotein E | Apoe | 0.75 | 2574 | 5906 | 2.72 | 2.64 |
| ubiquinol-cytochrome c reductase binding protein | Uqcrb | 0.73 | 2406 | 5907 | 1.23 | 1.53 |
| chemokine (C-X3-C motif) receptor 1 | Cx3cr1 | 0.73 | 1219 | 4537 | 1.55 | 1.61 |
| POU domain, class 2, transcription factor 2 | Pou2f2 | 0.73 | 2046 | 5528 | 2.24 | 2.23 |
| enolase 3, beta muscle | Eno3 | 0.73 | 1004 | 4068 | 1.58 | 1.61 |
| ATPase, Na+/K+ transporting, alpha 1 polypeptide | Atp1a1 | 0.72 | 3165 | 6202 | 1.61 | 1.90 |
| cystatin C | Cst3 | 0.72 | 8352 | 9821 | 2.71 | 3.51 |
| translocator protein | Tspo | 0.71 | 5997 | 8820 | 1.80 | 2.02 |
| coiled-coil domain containing 50 | Ccdc50 | 0.71 | 1306 | 4751 | 1.13 | 1.37 |
| triggering receptor expressed on myeloid cells-like 4 | Treml4 | 0.70 | 2013 | 5459 | 1.81 | 1.82 |

## Supplemental Table 3. Down-regulated genes in *Lal*$^{-/-}$ CD11c$^{+}$ cells (top 50)

| Genes | Symbol | LogFC | # of cells | | Expression | |
|---|---|---|---|---|---|---|
| | | | *Lal*$^{+/+}$ | *Lal*$^{-/-}$ | *Lal*$^{+/+}$ | *Lal*$^{-/-}$ |
| FBJ osteosarcoma oncogene | Fos | -2.17 | 8595 | 3549 | 3.09 | 1.55 |
| dual specificity phosphatase 1 | Dusp1 | -1.58 | 8798 | 6456 | 3.20 | 1.75 |
| ribosomal protein S18, pseudogene 6 | Gm10260 | -1.52 | 3736 | 70 | 2.23 | 0.47 |
| early growth response 1 | Egr1 | -1.38 | 3368 | 321 | 2.21 | 1.26 |
| predicted gene, 34084 | Gm34084 | -1.32 | 4162 | 30 | 1.95 | 1.04 |
| immunoglobulin kappa constant | Igkc | -1.27 | 1145 | 223 | 2.17 | 2.03 |
| myeloid-associated differentiation marker | Myadm | -1.21 | 7061 | 4385 | 2.27 | 1.28 |
| zinc finger protein 36 | Zfp36 | -1.19 | 9144 | 7735 | 2.68 | 1.59 |
| | Gm26532 | -1.19 | 5443 | 1784 | 1.98 | 1.18 |
| chemokine (C-C motif) ligand 5 | Ccl5 | -1.18 | 1406 | 436 | 3.14 | 2.53 |
| FBJ osteosarcoma oncogene B | Fosb | -1.13 | 2941 | 173 | 1.96 | 0.77 |
| colony stimulating factor 3 receptor (granulocyte) | Csf3r | -1.12 | 8487 | 7112 | 3.52 | 2.19 |
| DENN/MADD domain containing 4A | Dennd4a | -1.08 | 7357 | 4275 | 2.27 | 1.47 |
| Kruppel-like factor 6 | Klf6 | -1.05 | 9321 | 7725 | 2.52 | 1.58 |
| immediate early response 2 | Ier2 | -1.03 | 8658 | 7038 | 2.31 | 1.46 |
| jun B proto-oncogene | Junb | -1.00 | 10148 | 9097 | 3.14 | 2.13 |
| neutrophil cytosolic factor 2 | Ncf2 | -0.98 | 8803 | 8521 | 2.74 | 1.80 |
| TSC22 domain family, member 3 | Tsc22d3 | -0.98 | 9465 | 7495 | 2.56 | 1.63 |
| chemokine (C-X-C motif) receptor 4 | Cxcr4 | -0.97 | 6777 | 4258 | 2.08 | 1.31 |
| regulator of G-protein signaling | Rgs2 | -0.97 | 8895 | 8317 | 2.81 | 1.85 |
| jun D proto-oncogene | Jund | -0.97 | 10262 | 8732 | 2.84 | 1.85 |
| nuclear paraspeckle assembly transcript 1 (non-protein coding) | Neat1 | -0.96 | 9772 | 9346 | 3.04 | 2.16 |
| elongator complex protein 1 | Ikbkap | -0.96 | 4350 | 1597 | 1.93 | 1.20 |
| prostaglandin-endoperoxide synthase 2 | Ptgs2 | -0.95 | 1199 | 118 | 2.46 | 1.84 |
| BTG anti-proliferation factor 2 | Btg2 | -0.94 | 9020 | 7496 | 2.32 | 1.49 |
| fibrinogen-like protein 2 | Fgl2 | -0.94 | 7965 | 4908 | 2.83 | 2.10 |
| RAB11 family interacting protein 1 (class I) | Rab11fip1 | -0.94 | 6216 | 3322 | 2.09 | 1.42 |
| thioredoxin interacting protein | Txnip | -0.94 | 9419 | 6860 | 2.45 | 1.62 |
| ATPase, class VI, type 11B | Atp11b | -0.93 | 7620 | 5414 | 2.13 | 1.37 |
| insulin-like growth factor I receptor | Igf1r | -0.90 | 6580 | 3760 | 2.37 | 1.76 |
| interleukin 1 beta | Il1b | -0.89 | 8688 | 6920 | 3.66 | 2.59 |
| arginase type II | Arg2 | -0.87 | 5248 | 2412 | 2.14 | 1.65 |
| cytotoxic T lymphocyte-associated protein 2 alpha | Ctla2a | -0.87 | 2287 | 524 | 2.11 | 1.84 |
| arrestin domain containing 3 | Arrdc3 | -0.85 | 5007 | 2627 | 1.97 | 1.33 |
| immediate early response 5 | Ier5 | -0.85 | 9120 | 8059 | 2.40 | 1.59 |
| chemokine (C-C motif) ligand 6 | Ccl6 | -0.80 | 7579 | 7024 | 2.68 | 1.79 |
| junction adhesion molecule like | Jaml | -0.80 | 6351 | 4334 | 2.40 | 1.71 |
| myelin basic protein | Mbp | -0.80 | 6823 | 5382 | 1.99 | 1.28 |
| transmembrane and coiled coil domains 1 | Tmcc1 | -0.79 | 7641 | 6923 | 2.30 | 1.55 |
| selectin, lymphocyte | Sell | -0.78 | 7555 | 4215 | 2.25 | 1.85 |
| nuclear factor of kappa light polypeptide gene enhancer in B cells inhibitor, zeta | Nfkbiz | -0.77 | 4985 | 4145 | 1.93 | 1.15 |
| expressed sequence AI467606 | AI467606 | -0.77 | 6523 | 4063 | 1.87 | 1.29 |
| matrix metallopeptidase 9 | Mmp9 | -0.77 | 7119 | 3988 | 2.83 | 2.47 |
| nischarin | Nisch | -0.77 | 5804 | 4644 | 1.88 | 1.15 |
| zinc finger protein 36, C3H type-like 2 | Zfp36l2 | -0.77 | 9364 | 8093 | 2.40 | 1.71 |
| Kruppel-like factor 3 (basic) | Klf3 | -0.76 | 8164 | 6950 | 2.15 | 1.45 |
| CDC-like kinase 1 | Clk1 | -0.76 | 7611 | 6131 | 1.94 | 1.27 |
| colony stimulating factor 1 (macrophage) | Csf1 | -0.73 | 3845 | 1533 | 2.16 | 1.87 |
| membrane-spanning 4-domains, subfamily A, member 4B | Ms4a4b | -0.73 | 1724 | 906 | 2.44 | 1.23 |
| transient receptor potential cation channel, subfamily M, member 2 | Trpm2 | -0.73 | 3854 | 2491 | 1.93 | 1.17 |

## Supplemental Table 4. Up-regulated genes in Cluster 158 of *Lal*$^{-/-}$ CD11c$^{+}$ cells (top 50)

| Genes | Symbol | LogFC | # of cells | | Expression | |
|---|---|---|---|---|---|---|
| | | | *Lal*$^{+/+}$ | *Lal*$^{-/-}$ | *Lal*$^{+/+}$ | *Lal*$^{-/-}$ |
| chitinase-like 3 | Chil3 | 1.76 | 181 | 3551 | 1.48 | 2.09 |
| hypoxanthine guanine phosphoribosyl transferase | Hprt | 1.74 | 2 | 4717 | 0.93 | 1.66 |
| haptoglobin | Hp | 1.63 | 492 | 4748 | 1.34 | 2.39 |
| glutathione reductase | Gsr | 1.51 | 536 | 4903 | 1.04 | 2.11 |
| lectin, galactose binding, soluble 3 | Lgals3 | 1.25 | 1010 | 4964 | 2.06 | 3.29 |
| predicted pseudogene 10320 | Gm10320 | 1.18 | 2 | 4065 | 0.52 | 1.24 |
| ferritin heavy polypeptide 1 | Fth1 | 1.09 | 1072 | 4975 | 3.14 | 4.06 |
| coagulation factor X | F10 | 1.09 | 235 | 4097 | 0.95 | 1.40 |
| thioredoxin 1 | Txn1 | 1.08 | 905 | 4941 | 1.51 | 2.48 |
| transmembrane protein 14C | Tmem14c | 1.07 | 358 | 4607 | 0.91 | 1.48 |
| peroxiredoxin 5 | Prdx5 | 1.07 | 927 | 4887 | 1.83 | 2.72 |
| serum amyloid A 3 | Saa3 | 1.04 | 5 | 744 | 0.84 | 1.65 |
| superoxide dismutase 2, mitochondrial | Sod2 | 1.02 | 500 | 4431 | 1.02 | 1.57 |
| WAP four-disulfide core domain 17 | Wfdc17 | 1.02 | 105 | 2350 | 1.08 | 1.26 |
| secretory leukocyte peptidase inhibitor | Slpi | 0.96 | 229 | 3485 | 1.03 | 1.34 |
| formyl peptide receptor 2 | Fpr2 | 0.92 | 388 | 4391 | 1.24 | 1.58 |
| CD302 antigen | Cd302 | 0.85 | 387 | 4358 | 0.97 | 1.36 |
| chitinase-like 1 | Chil1 | 0.84 | 201 | 3155 | 1.11 | 1.35 |
| heme binding protein 1 | Hebp1 | 0.84 | 176 | 3596 | 0.93 | 1.21 |
| mitochondrial ribosomal protein L52 | Mrpl52 | 0.84 | 493 | 4531 | 1.04 | 1.46 |
| histocompatibility 2, class II antigen E beta | H2-Eb1 | 0.84 | 95 | 861 | 0.96 | 1.73 |
| transforming growth factor, beta induced | Tgfbi | 0.82 | 455 | 4000 | 1.30 | 1.66 |
| formyl peptide receptor 1 | Fpr1 | 0.82 | 72 | 2942 | 0.96 | 1.16 |
| expressed sequence AW112010 | AW112010 | 0.81 | 334 | 3594 | 1.09 | 1.39 |
| cathepsin C | Ctsc | 0.80 | 569 | 4410 | 1.28 | 1.66 |
| C-type lectin domain family 4, member e | Clec4e | 0.75 | 494 | 4389 | 1.24 | 1.51 |
| interferon activated gene 207 | Ifi207 | 0.75 | 62 | 3092 | 0.80 | 1.02 |
| cathepsin B | Cstb | 0.74 | 339 | 3418 | 0.98 | 1.30 |
| cathepsin D | Ctsd | 0.74 | 766 | 4546 | 1.34 | 1.72 |
| inhibitor of kappaB kinase epsilon | Ikbke | 0.73 | 39 | 3228 | 0.84 | 0.96 |
| CD14 antigen | Cd14 | 0.69 | 424 | 4169 | 1.19 | 1.38 |
| phospholipase A2, group VII (platelet-activating factor acetylhydrolase, plasma) | Pla2g7 | 0.69 | 914 | 4900 | 1.74 | 2.32 |
| inhibitor of DNA binding 2 | Id2 | 0.69 | 365 | 3552 | 1.23 | 1.42 |
| integrin alpha 4 | Itga4 | 0.69 | 977 | 4887 | 1.67 | 2.24 |
| placenta-specific 8 | Plac8 | 0.68 | 1001 | 4893 | 2.45 | 3.08 |
| protein tyrosine phosphatase, non-receptor type 1 | Ptpn1 | 0.68 | 969 | 4934 | 1.76 | 2.37 |
| guanylate binding protein 2 | Gbp2 | 0.67 | 111 | 2198 | 0.97 | 1.17 |
| allograft inflammatory factor 1 | Aif1 | 0.67 | 325 | 3361 | 1.21 | 1.39 |
| ribosomal protein S18 | Rps18 | 0.67 | 1056 | 4964 | 2.33 | 3.01 |
| ninjurin 1 | Ninj1 | 0.67 | 297 | 3366 | 0.98 | 1.19 |
| fatty acid binding protein 5, epidermal | Fabp5 | 0.67 | 134 | 2187 | 0.94 | 1.10 |
| ubiquitin C | Ubc | 0.66 | 928 | 4923 | 1.59 | 2.15 |
| peroxiredoxin 1 | Prdx1 | 0.66 | 998 | 4909 | 1.78 | 2.25 |
| selection and upkeep of intraepithelial T cells 3 | Skint3 | 0.66 | 0 | 2459 | 0 | 0.97 |
| complement component 3 | C3 | 0.66 | 348 | 3913 | 1.09 | 1.27 |
| lectin, galactose binding, soluble 1 | Lgals1 | 0.66 | 550 | 3674 | 1.21 | 1.50 |
| C-type lectin domain family 4, member n | Clec4n | 0.65 | 133 | 2502 | 0.90 | 1.11 |
| uridine phosphorylase 1 | Upp1 | 0.65 | 36 | 2069 | 0.91 | 1.10 |
| interferon activated gene 204 | Ifi204 | 0.63 | 102 | 2958 | 0.87 | 0.99 |
| ubiquinol-cytochrome c reductase binding protein | Uqcrb | 0.61 | 730 | 4667 | 1.15 | 1.54 |

## Supplemental Table 5. Down-regulated genes in Cluster 158 of *Lal*$^{-/-}$ CD11c$^{+}$ cells (top 50)

| Genes | Symbol | LogFC | # of cells | | Expression | |
|---|---|---|---|---|---|---|
| | | | *Lal*$^{+/+}$ | *Lal*$^{-/-}$ | *Lal*$^{+/+}$ | *Lal*$^{-/-}$ |
| heat shock protein 1B | Hspa1b | -1.90 | 828 | 1523 | 2.30 | 1.01 |
| heat shock protein 1A | Hspa1a | -1.89 | 780 | 1128 | 2.22 | 0.96 |
| transferrin | Trf | -1.88 | 1036 | 1856 | 2.22 | 0.91 |
| ribosomal protein S18, pseudogene 6 | Gm10260 | -1.86 | 985 | 54 | 1.83 | 0.47 |
| B cell leukemia/lymphoma 2 related protein A1d | Bcl2a1d | -1.51 | 961 | 1627 | 1.82 | 0.80 |
| eosinophil-associated, ribonuclease A family, member 1 | Ear1 | -1.50 | 796 | 6 | 1.58 | 0.67 |
| CD74 antigen (invariant polypeptide of major histocompatibility complex, class II antigen-associated) | Cd74 | -1.43 | 938 | 2806 | 3.19 | 2.01 |
| immunoglobulin heavy constant mu | Ighm | -1.23 | 816 | 652 | 1.48 | 0.79 |
| phospholipid transfer protein | Pltp | -1.10 | 830 | 1616 | 1.66 | 1.04 |
| TSC22 domain family, member 3 | Tsc22d3 | -1.08 | 1018 | 3572 | 1.93 | 1.08 |
| FBJ osteosarcoma oncogene | Fos | -1.06 | 677 | 1383 | 1.65 | 0.98 |
| jun D proto-oncogene | Jund | -1.05 | 1042 | 4212 | 2.25 | 1.31 |
| histocompatibility 2, class II antigen A, beta 1 | H2-Ab1 | -1.00 | 402 | 1229 | 2.41 | 1.76 |
| dual specificity phosphatase 1 | Dusp1 | -0.95 | 849 | 2816 | 1.74 | 1.04 |
| histocompatibility 2, class II antigen A, alpha | H2-Aa | -0.91 | 387 | 1122 | 2.40 | 1.85 |
| inactive X specific transcripts | Xist | -0.91 | 342 | 905 | 2.45 | 1.75 |
| ATPase, class VI, type 11B | Atp11b | -0.85 | 919 | 3213 | 1.77 | 1.11 |
| chemokine (C-C motif) ligand 9 | Ccl9 | -0.84 | 815 | 2233 | 1.74 | 1.20 |
| | AC160336.1 | -0.84 | 934 | 3590 | 1.78 | 1.12 |
| polymerase (RNA) II (DNA directed) polypeptide L | Polr2l | -0.81 | 828 | 2899 | 1.61 | 0.99 |
| chemokine (C-X-C motif) receptor 4 | Cxcr4 | -0.80 | 870 | 2479 | 1.50 | 0.96 |
| BTG anti-proliferation factor 2 | Btg2 | -0.78 | 999 | 4172 | 1.87 | 1.20 |
| CD83 antigen | Cd83 | -0.78 | 551 | 624 | 1.29 | 0.87 |
| thioredoxin interacting protein | Txnip | -0.77 | 862 | 2952 | 1.55 | 0.95 |
| lysosomal acid lipase A | Lipa | -0.75 | 727 | 1659 | 1.27 | 0.74 |
| zinc finger protein 36, C3H type-like 2 | Zfp36l2 | -0.75 | 1057 | 4441 | 2.15 | 1.48 |
| Kruppel-like factor 6 | Klf6 | -0.74 | 980 | 3989 | 1.79 | 1.16 |
| | Gm47283 | -0.73 | 614 | 914 | 1.17 | 0.68 |
| hes family bHLH transcription factor 1 | Hes1 | -0.72 | 744 | 2311 | 1.52 | 1.02 |
| serine/threonine kinase 24 | Stk24 | -0.70 | 1014 | 4270 | 1.80 | 1.17 |
| myelin basic protein | Mbp | -0.68 | 965 | 3639 | 1.65 | 1.11 |
| DENN/MADD domain containing 4A | Dennd4a | -0.67 | 643 | 1625 | 1.30 | 0.82 |
| terminal nucleotidyltransferase 5A | Fam46a | -0.67 | 981 | 4046 | 1.86 | 1.30 |
| zinc finger protein 36 | Zfp36 | -0.67 | 981 | 4116 | 1.80 | 1.21 |
| RAB11 family interacting protein 1 (class I) | Rab11fip1 | -0.67 | 594 | 1131 | 1.20 | 0.74 |
| CD36 molecule | Cd36 | -0.66 | 736 | 1800 | 1.56 | 1.23 |
| FXYD domain-containing ion transport regulator 5 | Fxyd5 | -0.65 | 1069 | 4848 | 2.63 | 1.95 |
| transmembrane and coiled coil domains 1 | Tmcc1 | -0.65 | 995 | 4167 | 1.91 | 1.33 |
| | Gm26532 | -0.64 | 539 | 810 | 1.16 | 0.71 |
| apolipoprotein E | Apoe | -0.62 | 1008 | 4440 | 3.72 | 2.89 |
| ribosomal protein S18, pseudogene 5 | Gm11361 | -0.62 | 495 | 85 | 1.02 | 0.56 |
| salt inducible kinase 1 | Sik1 | -0.61 | 655 | 1500 | 1.19 | 0.78 |
| leukocyte immunoglobulin-like receptor, subfamily A (with TM domain), member 5 | Lilra5 | -0.61 | 848 | 3094 | 1.71 | 1.24 |
| myeloid-associated differentiation marker | Myadm | -0.61 | 716 | 2232 | 1.28 | 0.82 |
| jun B proto-oncogene | Junb | -0.61 | 1014 | 4459 | 2.08 | 1.54 |
| adhesion G protein-coupled receptor E5 | Adgre5 | -0.60 | 1068 | 4870 | 2.57 | 1.95 |
| cysteine-rich protein 1 (intestinal) | Crip1 | -0.58 | 1007 | 4160 | 2.52 | 1.97 |
| complement component factor h | Cfh | -0.58 | 689 | 1569 | 1.27 | 0.97 |
| AHNAK nucleoprotein (desmoyokin) | Ahnak | -0.58 | 926 | 3720 | 1.89 | 1.44 |
| cDNA sequence AY036118 | AY036118 | -0.58 | 1054 | 4832 | 2.36 | 1.80 |

## Supplemental Table 6. Up-regulated genes in Cluster 0236 of *Lal*$^{-/-}$ CD11c$^{+}$ cells (top 50)

| Genes | Symbol | LogFC | # of cells | | Expression | |
|---|---|---|---|---|---|---|
| | | | *Lal*$^{+/+}$ | *Lal*$^{-/-}$ | *Lal*$^{+/+}$ | *Lal*$^{-/-}$ |
| interferon induced transmembrane protein 1 | Ifitm1 | 2.24 | 4747 | 3935 | 2.46 | 4.29 |
| WAP four-disulfide core domain 17 | Wfdc17 | 2.17 | 2626 | 3694 | 2.53 | 3.77 |
| glutathione reductase | Gsr | 2.14 | 1192 | 3864 | 1.81 | 2.70 |
| lectin, galactose binding, soluble 3 | Lgals3 | 1.99 | 2745 | 3902 | 2.12 | 3.32 |
| neutrophilic granule protein | Ngp | 1.99 | 121 | 823 | 1.78 | 2.58 |
| lipocalin 2 | Lcn2 | 1.69 | 1819 | 2440 | 2.31 | 3.04 |
| WAP four-disulfide core domain 21 | Wfdc21 | 1.55 | 3384 | 3366 | 2.44 | 3.41 |
| interferon induced transmembrane protein 6 | Ifitm6 | 1.51 | 711 | 1561 | 1.81 | 2.37 |
| insulin-like growth factor binding protein 6 | Igfbp6 | 1.41 | 581 | 2052 | 1.91 | 2.22 |
| prokineticin 2 | Prok2 | 1.15 | 42 | 852 | 1.88 | 2.16 |
| transmembrane protein 14C | Tmem14c | 1.14 | 444 | 2092 | 1.72 | 1.81 |
| interferon induced transmembrane protein 3 | Ifitm3 | 1.13 | 2983 | 3304 | 2.04 | 2.58 |
| translocator protein | Tspo | 1.12 | 3176 | 3335 | 2.02 | 2.59 |
| resistin like gamma | Retnlg | 1.12 | 4842 | 3110 | 3.18 | 3.89 |
| uridine phosphorylase 1 | Upp1 | 1.03 | 226 | 1477 | 1.88 | 1.86 |
| placenta-specific 8 | Plac8 | 1.03 | 1295 | 2105 | 2.18 | 2.26 |
| hypoxanthine guanine phosphoribosyl transferase | Hprt | 1.02 | 1 | 1079 | 1.28 | 1.89 |
| RIKEN cDNA 1600014C10 gene | 1600014C10Rik | 1.01 | 759 | 1593 | 1.80 | 2.01 |
| interferon induced transmembrane protein 2 | Ifitm2 | 0.98 | 6033 | 4042 | 2.92 | 3.82 |
| STEAP family member 4 | Steap4 | 0.95 | 900 | 2043 | 2.12 | 2.09 |
| leucine-rich alpha-2-glycoprotein 1 | Lrg1 | 0.93 | 4264 | 3732 | 2.51 | 3.08 |
| predicted pseudogene 10320 | Gm10320 | 0.89 | 8 | 1258 | 1.62 | 1.64 |
| cytochrome c oxidase I, mitochondrial | mt-Co1 | 0.86 | 4405 | 3699 | 2.43 | 2.97 |
| predicted gene, 35082 | Gm35082 | 0.84 | 287 | 1439 | 1.88 | 1.74 |
| S100 calcium binding protein A9 (calgranulin B) | S100a9 | 0.83 | 6689 | 4014 | 5.36 | 6.17 |
| destrin | Dstn | 0.81 | 729 | 1699 | 1.79 | 1.84 |
| catenin (cadherin associated protein), beta 1 | Ctnnb1 | 0.81 | 1960 | 2685 | 1.90 | 2.02 |
| stefin A2 like 1 | Stfa2l1 | 0.81 | 1978 | 1993 | 2.66 | 2.91 |
| chitinase-like 1 | Chil1 | 0.80 | 2924 | 3087 | 2.03 | 2.28 |
| sorcin | Sri | 0.76 | 1482 | 2263 | 1.91 | 2.00 |
| lymphocyte antigen 6 complex, locus G | Ly6g | 0.73 | 245 | 880 | 1.82 | 1.91 |
| ATP synthase, H+ transporting, mitochondrial F0 complex, subunit D | Atp5h | 0.72 | 975 | 1902 | 1.77 | 1.75 |
| S100 calcium binding protein A8 (calgranulin A) | S100a8 | 0.71 | 6680 | 3953 | 5.18 | 5.62 |
| glyceraldehyde-3-phosphate dehydrogenase | Gapdh | 0.69 | 4140 | 3422 | 2.18 | 2.55 |
| ferritin heavy polypeptide 1 | Fth1 | 0.66 | 6825 | 4073 | 3.74 | 4.26 |
| fatty acid binding protein 5, epidermal | Fabp5 | 0.66 | 731 | 1406 | 1.82 | 1.81 |
| Fc receptor, IgE, high affinity I, gamma polypeptide | Fcer1g | 0.64 | 6291 | 4042 | 2.81 | 3.37 |
| neutrophil cytosolic factor 4 | Ncf4 | 0.63 | 4763 | 3749 | 2.23 | 2.58 |
| ribonuclease, RNase A family, 6 | Rnase6 | 0.60 | 112 | 692 | 1.87 | 1.80 |
| cytochrome c oxidase subunit 6C | Cox6c | 0.60 | 1594 | 2134 | 1.86 | 1.84 |
| cystatin C | Cst3 | 0.59 | 5504 | 3914 | 2.51 | 2.86 |
| ectonucleoside triphosphate diphosphohydrolase 3 | Entpd3 | 0.58 | 247 | 736 | 1.82 | 1.86 |
| CD52 antigen | Cd52 | 0.57 | 6268 | 3995 | 2.87 | 3.36 |
| BRD8 domain containing | 4933408B17Rik | 0.56 | 21 | 773 | 1.84 | 1.54 |
| refilin B | Rflnb | 0.56 | 694 | 1304 | 1.80 | 1.72 |
| guanine nucleotide binding protein (G protein), gamma 12 | Gng12 | 0.55 | 1643 | 2113 | 1.82 | 1.80 |
| X-box binding protein 1 | Xbp1 | 0.55 | 1012 | 1428 | 1.86 | 1.85 |
| chitinase-like 3 | Chil3 | 0.55 | 52 | 513 | 1.82 | 1.75 |
| H3.3 histone A | H3f3a | 0.53 | 6594 | 4060 | 3.08 | 3.59 |
| cyclin dependent kinase inhibitor 2D | Cdkn2d | 0.53 | 1631 | 2036 | 1.91 | 1.88 |

## Supplemental Table 7. Down-regulated genes in Cluster 0236 of *Lal*$^{-/-}$ CD11c$^{+}$ cells (top 50)

| Genes | Symbol | LogFC | # of cells | | Expression | |
|---|---|---|---|---|---|---|
| | | | *Lal*$^{+/+}$ | *Lal*$^{-/-}$ | *Lal*$^{+/+}$ | *Lal*$^{-/-}$ |
| FBJ osteosarcoma oncogene | Fos | -2.03 | 6777 | 1869 | 3.43 | 2.01 |
| early growth response 1 | Egr1 | -1.61 | 2856 | 110 | 2.35 | 1.92 |
| predicted gene, 34084 | Gm34084 | -1.58 | 3536 | 12 | 2.07 | 1.82 |
| FBJ osteosarcoma oncogene B | Fosb | -1.36 | 2309 | 28 | 2.15 | 1.77 |
| dual specificity phosphatase 1 | Dusp1 | -1.28 | 6922 | 3280 | 3.55 | 2.38 |
| | Gm26532 | -1.17 | 4128 | 808 | 2.20 | 1.71 |
| immediate early response 2 | Ier2 | -1.13 | 5900 | 2172 | 2.62 | 1.88 |
| prostaglandin-endoperoxide synthase 2 | Ptgs2 | -1.11 | 1083 | 98 | 2.54 | 2.03 |
| myeloid-associated differentiation marker | Myadm | -1.08 | 5442 | 1834 | 2.52 | 1.87 |
| zinc finger protein 36 | Zfp36 | -1.07 | 6725 | 3046 | 3.02 | 2.13 |
| nuclear factor of kappa light polypeptide gene enhancer in B cells inhibitor, zeta | Nfkbiz | -0.99 | 3543 | 902 | 2.19 | 1.69 |
| neutrophil cytosolic factor 2 | Ncf2 | -0.99 | 6723 | 3234 | 2.99 | 2.16 |
| elongator complex protein 1 | Ikbkap | -0.94 | 3750 | 941 | 2.05 | 1.57 |
| regulator of G-protein signaling | Rgs2 | -0.93 | 6707 | 3229 | 3.10 | 2.28 |
| cytotoxic T lymphocyte-associated protein 2 alpha | Ctla2a | -0.91 | 1232 | 131 | 2.34 | 2.04 |
| ATPase, class VI, type 11B | Atp11b | -0.90 | 5191 | 1701 | 2.40 | 1.91 |
| myelin basic protein | Mbp | -0.90 | 4635 | 1396 | 2.25 | 1.81 |
| chemokine (C-X-C motif) receptor 4 | Cxcr4 | -0.88 | 4674 | 1501 | 2.36 | 1.92 |
| BTG anti-proliferation factor 2 | Btg2 | -0.85 | 5914 | 2625 | 2.60 | 1.98 |
| Kruppel-like factor 6 | Klf6 | -0.85 | 6420 | 3079 | 2.87 | 2.17 |
| nuclear paraspeckle assembly transcript 1 (non-protein coding) | Neat1 | -0.84 | 6935 | 3761 | 3.48 | 2.66 |
| DENN/MADD domain containing 4A | Dennd4a | -0.83 | 5494 | 2281 | 2.54 | 1.98 |
| tribbles pseudokinase 1 | Trib1 | -0.82 | 2558 | 647 | 2.10 | 1.69 |
| transmembrane and coiled coil domains 1 | Tmcc1 | -0.81 | 5561 | 2360 | 2.53 | 1.98 |
| | AC160336.1 | -0.79 | 3673 | 1261 | 2.21 | 1.78 |
| Dr1 associated protein 1 (negative cofactor 2 alpha) | Drap1 | -0.75 | 2168 | 390 | 1.95 | 1.63 |
| arrestin domain containing 3 | Arrdc3 | -0.74 | 3958 | 1274 | 2.15 | 1.81 |
| activating transcription factor 3 | Atf3 | -0.74 | 1988 | 290 | 1.98 | 1.84 |
| RAB11 family interacting protein 1 (class I) | Rab11fip1 | -0.73 | 5051 | 1987 | 2.27 | 1.82 |
| nischarin | Nisch | -0.73 | 4073 | 1571 | 2.17 | 1.74 |
| colony stimulating factor 3 receptor (granulocyte) | Csf3r | -0.72 | 7048 | 3997 | 3.85 | 3.03 |
| jun B proto-oncogene | Junb | -0.71 | 7002 | 3889 | 3.63 | 2.86 |
| transient receptor potential cation channel, subfamily M, member 2 | Trpm2 | -0.69 | 3266 | 1101 | 2.06 | 1.66 |
| CDC-like kinase 1 | Clk1 | -0.68 | 4826 | 1954 | 2.24 | 1.82 |
| TSC22 domain family, member 3 | Tsc22d3 | -0.68 | 6445 | 3304 | 2.90 | 2.28 |
| immediate early response 5 | Ier5 | -0.68 | 6328 | 3164 | 2.73 | 2.15 |
| Kruppel-like factor 3 (basic) | Klf3 | -0.67 | 5356 | 2366 | 2.42 | 1.96 |
| zinc finger protein 36, C3H type-like 2 | Zfp36l2 | -0.67 | 6131 | 2903 | 2.66 | 2.13 |
| chemokine (C-C motif) ligand 6 | Ccl6 | -0.67 | 5973 | 2573 | 2.89 | 2.45 |
| RIKEN cDNA 1700020D05 gene | 1700020D05 Rik | -0.66 | 1188 | 2 | 1.82 | 1.64 |
| guanine nucleotide binding protein (G protein), gamma 11 | Gng11 | -0.66 | 834 | 67 | 2.13 | 1.87 |
| jun D proto-oncogene | Jund | -0.66 | 6773 | 3642 | 3.16 | 2.51 |
| platelet factor 4 | Pf4 | -0.65 | 915 | 92 | 2.21 | 2.19 |
| arginase type II | Arg2 | -0.65 | 4702 | 1976 | 2.22 | 1.80 |
| nucleosome assembly protein 1-like 1 | Nap1l1 | -0.63 | 2902 | 1011 | 2.16 | 1.83 |
| ST3 beta-galactoside alpha-2,3-sialyltransferase 6 | St3gal6 | -0.62 | 2106 | 522 | 1.93 | 1.63 |
| Hist1h1cR1 erythroid cis-regulatory module | Hist1h1c | -0.61 | 2700 | 991 | 2.20 | 1.86 |
| activating transcription factor 4 | Atf4 | -0.61 | 2120 | 568 | 1.97 | 1.68 |
| mitogen-activated protein kinase kinase kinase 5 | Map3k5 | -0.60 | 2449 | 697 | 1.96 | 1.69 |
| thioredoxin interacting protein | Txnip | -0.59 | 6356 | 3232 | 2.79 | 2.27 |
